# Hidden hematological, biochemical and immune costs of asymptomatic malaria infections in semi-wild chimpanzees

**DOI:** 10.1371/journal.ppat.1014287

**Published:** 2026-06-23

**Authors:** Anais Nowakowski, Eric Elguero, Katie Patterson, Anne Boissière, Fanny Degrugillier, Christine Sidobre, Celine Arnathau, Pauline Grentzinger, Eric Willaume, Arthur M. Talman, Benoit Malleret, Larson Boundenga, Barthelemy Ngoubangoye, Franck Prugnolle, Samuel C. Wassmer, Virginie Rougeron

**Affiliations:** 1 REHABS, International Research Laboratory, CNRS-NMU, George Campus, Nelson Mandela University, George, South Africa; 2 Laboratory MIVEGEC, University of Montpellier, CNRS, IRD, Montpellier, France; 3 Department of Infection Biology, London School of Hygiene & Tropical Medicine, London, United Kingdom; 4 Laboratory Animal, Santé, Territoire, Risque, Ecosystème (ASTRE), UMR 117, Campus International de Baillarguet, CIRAD, Montpellier, France; 5 Sodepal, La Lékédi Park, Bakoumba, Gabon; 6 Department of Microbiology and Immunology, Immunology Translational Research Programme, Yong Loo Lin School of Medicine, Immunology Programme, Life Sciences Institute, National University of Singapore, Singapore, Singapore; 7 Singapore Immunology Network (SIgN), Agency for Science & Technology, Singapore, Singapore; 8 CIRMF, Centre Interdisciplinaire de Recherches Médicales de Franceville, Franceville, Gabon; 9 Department of Anthropology, Durham University, Durham, United Kingdom; 10 Sustainability Research Unit, George Campus, Nelson Mandela University, George, South Africa; Burnet Institute, AUSTRALIA

## Abstract

The health consequences of *Plasmodium* infections in wild great apes, particularly in asymptomatic animals, remain poorly understood. This study investigated the hematological and immune impacts of natural malaria infections in 27 semi-wild chimpanzees (*Pan troglodytes troglodytes*) from Gabon. Using PCR and qPCR to identify infected individuals, and MinION sequencing to determine the *Plasmodium* species involved, results showed a 48.15% overall *Plasmodium* infection rate, with frequent multi-species co-infections involving *Plasmodium gaboni*, *Plasmodium reichenowi*, and *Plasmodium vivax-like* parasites. In addition, infected chimpanzees were younger than non-infected individuals, although no significant association was detected between age and parasitemia levels, and this interpretation should be considered cautiously given the limited number of juvenile animals included in the study. Multi-species infections, particularly triple infections involving *P. vivax-like* parasites, were associated with higher parasitemia levels. Profiling of 15 hematological markers and 8 cytokines/chemokines known to be associated with malarial infections in humans revealed significant alterations in infected chimpanzees, including elevated urea, reduced creatinine, and increased systemic concentrations of pro-inflammatory (TNF, IL-1β, CCL3) and anti-inflammatory (IL-10) cytokines. *Ex vivo* PBMC stimulation yielded higher IL-10 in infected than non-infected individuals, indicating a regulatory-skewed cytokine response at the time of sampling. These results suggest that malaria in chimpanzees is associated with systemic immune modulation and accompanied by signs of physiological stress, including potential renal dysfunction. This study challenges the assumption that chronic *Plasmodium* infections are entirely benign in great apes and highlights the need to integrate immunological health indicators into conservation strategies. Broader immune profiling and longitudinal studies will be essential in the future to assess long-term health outcomes and resilience in these endangered populations.

## Author summary

Malaria parasites infect humans and many other primates, including African great apes such as chimpanzees. Although infections are common in these animals, they are generally assumed to be harmless because obvious disease symptoms are rarely observed. However, little is known about whether these infections affect ape health in more subtle ways. In this study, we investigated the physiological consequences of natural malaria infections in 27 semi-wild chimpanzees living in Gabon. Using molecular methods to identify parasite species and a panel of blood and immune markers, infected and non-infected individuals were compared. Nearly half of the chimpanzees carried malaria parasites, often with multiple parasite species infecting the same individual. Infected chimpanzees showed measurable changes in several physiological indicators, including kidney-related biochemical markers and immune signaling molecules involved in inflammation and immune regulation. Infected chimpanzees were generally younger than non-infected individuals, although no significant relationship was detected between age and parasite density. Because very few juvenile individuals were included in the study, larger longitudinal studies will be necessary to better understand age-related malaria dynamics in African great apes. Although the chimpanzees did not show obvious clinical symptoms, our results suggest that malaria infections may still impose hidden physiological costs. These findings challenge the idea that malaria is completely benign in great apes and highlight the importance of monitoring infectious diseases when assessing the health and conservation of wild primate populations.

## Introduction

Malaria is a vector-borne disease caused by protozoan parasites of the genus *Plasmodium* [1]. Six species are currently recognized as infecting humans: *Plasmodium falciparum*, *Plasmodium vivax*, *Plasmodium malariae*, *Plasmodium ovale wallikeri*, *Plasmodium ovale curtisi*, and *Plasmodium knowlesi*. African great apes are similarly natural hosts to several *Plasmodium* species closely related to those infecting humans. These parasites are divided into two subgenera: (i) *Plasmodium*, which includes all human-infecting species except *P. falciparum*, and three species infecting apes, *Plasmodium vivax*-like, *Plasmodium malariae*-like, and *Plasmodium ovale*-like; and (ii) *Laverania*, which includes *P. falciparum* and six ape-specific species, *Plasmodium praefalciparum*, *Plasmodium reichenowi*, *Plasmodium billcollinsi*, *Plasmodium blacklocki*, *Plasmodium adleri*, and *Plasmodium gaboni*, that infect chimpanzees, gorillas, and/or bonobos [2–4]. Similar to human malaria parasites, *Plasmodium* species infecting African great apes are transmitted by mosquitoes of the genus *Anopheles*. However, transmission in forest ecosystems appears to involve distinct mosquito species adapted to sylvatic environments rather than the primary human malaria vectors such as *Anopheles gambiae* or *Anopheles funestus* [5–7]. Phylogenetic and population genetic studies have further shown that the diversity of ape *Plasmodium* parasites represents the evolutionary reservoir from which some human malaria parasites, including *Plasmodium falciparum*, originated through historical host-switch events [8–12]. African great apes, therefore, represent natural hosts for a diverse assemblage of *Plasmodium* parasites and may constitute sylvatic reservoirs for these lineages. However, current evidence suggests that most ape *Plasmodium* parasites circulate primarily within wildlife transmission cycles, and their role in sustaining malaria transmission in human populations remains limited.

While the health impacts of *P. falciparum* and *P. vivax* infections in humans are well documented, the consequences of *Plasmodium* infections in great apes currently remain poorly understood. This knowledge gap largely stems from the logistical challenges of monitoring natural infections in non-human primates (NHPs), including difficulties in sampling protected species and limited access to field-based laboratory facilities. Although some studies suggest that these infections are often asymptomatic in great apes, others have reported malaria-related clinical signs in chimpanzees, indicating potential variability in disease outcomes [3,13–20]. Historically, the first documented descriptions of malaria parasite infections in African great apes date back to the 1910s-20s [21–23]. Rodhain’s studies reported a lack of illness signs in chimpanzees (*Pan troglodytes troglodytes*) infected with either great ape or human *Plasmodium* strains [21]. Conversely, one study described a juvenile chimpanzee infected with *P. falciparum* and *P. vivax* that had fever and loss of appetite [22]. Despite limited and sometimes conflicting evidence, the prevailing view was not that chimpanzees fail to get infected, but that naturally acquired *Plasmodium* infections in chimpanzees are usually subclinical, with overt malaria illness considered rare in the wild. It was only decades later that sporadic reports began to document *Plasmodium* infections in chimpanzees housed in sanctuaries or captive settings, though these accounts also yielded inconsistent conclusions. For example, Hayakawa et al. (2008, 2009) reported chronic *P. malariae*-like infections in two captive chimpanzees in Japan who remained asymptomatic for over 30 years [24,25]. Similarly, Krief et al. (2010) reported *P. falciparum* infections in bonobos from the Democratic Republic of Congo without any clinical signs [26]. Conversely, other studies have documented symptoms in African great apes, such as Tarello (2005), who reported the death of a one-year-old chimpanzee infected with *P. reichenowi*, although the exact cause of death remained unclear [27].

To date, the only study to our knowledge documenting clinical signs associated with *Plasmodium* infection in African great apes is that of Herbert et al. [20], which reported anemia and hyperthermia in a juvenile chimpanzee naturally infected with *P. reichenowi* in Gabon. This case suggested that, under certain conditions, *Plasmodium* infections may have measurable health impacts (i.e., fever and strong anemia). However, it remains the sole documented instance of apparent malaria-associated pathology in chimpanzees, and no subsequent studies have definitively addressed the clinical consequences of natural infections in wild great apes. As a result, the research community remains divided (among others, [3,13,28]). Given the paucity of systematic clinical monitoring, current evidence cannot resolve the full spectrum of outcomes. Rather than a simple “severe vs. innocuous” dichotomy, effects likely vary with age and exposure (acquired immunity), parasite lineage and parasitemia, host species/individual condition, and co-infections/nutritional stress, so mild or subclinical impacts may be common yet under-detected in cross-sectional studies.

Given the ongoing debate surrounding the health impacts of malaria in African great apes, this study aimed to assess the hematological and immune consequences of natural *Plasmodium* infections in chimpanzees in Gabon. In humans, various hematological parameters are well-established indicators of malaria severity and parasite virulence during the asexual blood stage. Infections are typically associated with reduced platelet, leukocyte, lymphocyte, red blood cell, hematocrit and hemoglobin counts, as well as elevated monocyte and neutrophil counts [29]. Because malaria also frequently affects the kidneys and liver in humans, renal (urea, creatinine) and hepatic (ALAT, ASAT, GGT) biochemical markers, along with lipid metabolism markers (cholesterol, triglycerides), were included as key indicators of organ function and metabolic status [30,31]. Additionally, immune responses to *P. falciparum* in humans are characterized by the secretion of both pro- and anti-inflammatory cytokines to respectively control parasitemia and modulate immunopathology [32]. Based on these observations, we selected a panel of 15 hematological biomarkers (described above), as well as eight cytokine and chemokine markers selected based on their known role in malarial infections in humans (TNF, IL-6, IFN-γ, IL-1β, IL-4, CCL3/MIP-1α, CCL5/RANTES, and IL-10), for analysis in infected and non-infected chimpanzees. This comprehensive dataset enables the first detailed assessment of the hematological, biochemical and immunological effects of *Plasmodium* infection in semi-wild chimpanzees, providing meaningful comparisons with human malaria for the first time.

## Results

### Study population description

A total of 27 chimpanzees were included in this study. The sampled population comprised both males and females spanning a broad age range, from juveniles to older adults. Individuals originated from the same geographical region in Gabon and were sampled under comparable environmental conditions, allowing a cross-sectional comparison of infection status and physiological parameters. None of the animals displayed overt clinical signs of disease at the time of sampling. Malaria parasites are known to circulate endemically in wild African ape populations in this region [2,11,33], providing a natural setting to investigate host physiological responses to *Plasmodium* infections.

### *Plasmodium* species identification from MinION reads and specific *P. vivax-like* PCR

**Fig 1:**
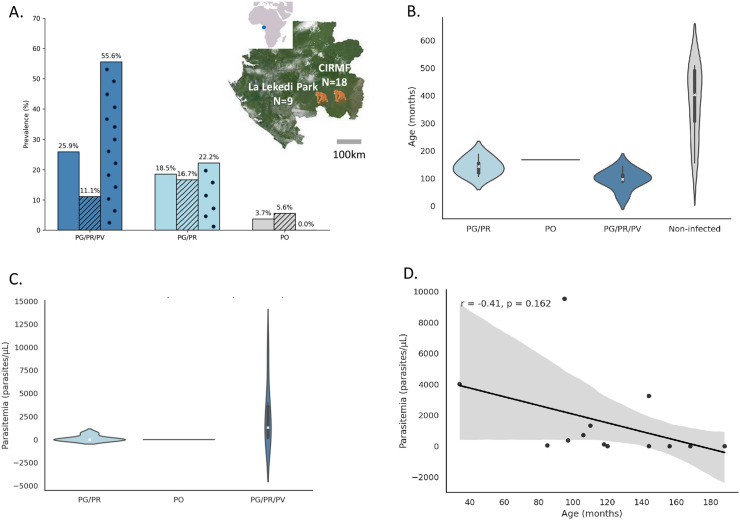
Geographic origin, *Plasmodium* prevalence, age distribution, and parasitemia levels in 27 chimpanzees from Gabon. **(A)** Geographic origin of sampled chimpanzees. Animals were sampled from two sites in Gabon: the Centre Interdisciplinaire de Recherches Médicales de Franceville (CIRMF; *N* = 18) and La Lékédi Park (*N* = 9). The bar plot shows the prevalence of each infection category across the full cohort (solid bars) and separately for each sampling site. Bars with diagonal stripes represent CIRMF, whereas bars with dotted patterns represent La Lekedi Park. Infection categories include PG/PR/PV (*P. gaboni*, *P. reichenowi*, *P. vivax*-like; n = 7), PG/PR (*P. gaboni*, *P. reichenowi*; n = 5), and PO (*P. ovale*-like; n = 1). Percentages are shown above each bar. Non-infected individuals are also shown (n = 14). Base maps of Africa and Gabon were obtained from Wikimedia Commons (https://commons.wikimedia.org/wiki/File:BlankMap-Africa.svg; https://commons.wikimedia.org/wiki/File:Gabon_sat.png) and modified by the authors. **(B)** Age distribution (in months) by infection status: non-infected (n = 14; light grey), PG/PR-infected (n = 5; light blue), PG/PR/PV-infected (n = 7; steel blue), and PO-infected (n = 1; dark grey). Violin plots display full distributions with internal boxplots showing medians and interquartile ranges; the PO-infected animal is represented by a single point (*n* = 1). **(C)** Parasitemia levels (parasites/μL of blood) by infection group. Violin plots show the distribution of parasitemia in PG/PR-, PO-, and PG/PR/PV-infected chimpanzees, with greater variability observed in the PG/PR/PV group. **(D)** Correlation between age and parasitemia in infected chimpanzees. Each point represents one animal. A moderate negative correlation is observed (r = –0.41, *p* = 0.162), suggesting younger animals tend to have higher parasitemia. The regression line is shown in black with a shaded 95% confidence interval.

In this study, we screened 27 chimpanzee blood samples for *Plasmodium* infection using nested PCR amplification of the *Plasmodium* cytochrome-b (cyt-b) gene combined with *P. vivax-like* specific PCR to determine infection status. Only samples that tested positive for *Plasmodium* by both nested PCR and quantitative PCR (qPCR) were subsequently subjected to MinION sequencing. For each sequenced sample, a random subset of 10,000 reads was analyzed, and a threshold of 300 reads (3%) was applied to distinguish true positives from background noise. This threshold accounts for the relatively high error rates associated with nanopore sequencing (10–15%), which aligns with prior studies in clinical diagnostics and metagenomics that use similar cutoffs to filter out low-confidence detections [29,30]. Based on this threshold, all *Plasmodium*-positive identifications were of high confidence and supported by a robust read count. Results revealed that co-infections involving *P. gaboni* (PG) and *P. reichenowi* (PR) were frequent, and only one animal had a mono-infection with a *P. ovale*-like (PO) parasite (Table A in S1 File). To overcome the limited sensitivity of cyt-b for detecting *P. vivax-like* (PV) parasites, we additionally performed a PCR targeting the mitochondrial cytochrome oxidase I (cox1) gene (Liu et al.) [34,35], and combined these results to estimate an overall *Plasmodium* infection prevalence of 48.15% (13/27; all species pooled; Fig 1A). Among the infected chimpanzees, 25.91% (7/27) had tri-infections with *P. gaboni*, *P. reichenowi*, and *P. vivax*-like (PG/PR/PV), 18.54% (5/27) had bi-infections with *P. gaboni* and *P. reichenowi* (PG/PR), and 3.7% (1/27) had a mono-infection with *P. ovale*-like (Fig 1A). Overall, 13 of the 27 chimpanzees were infected with *Plasmodium* parasites, while 14 were classified as non-infected based on molecular screening. Infected individuals were detected at both sampling sites. Among the 18 chimpanzees sampled at CIRMF, 6 were infected, and 12 were non-infected, whereas among the 9 individuals sampled at La Lekedi Park, 7 were infected and 2 were non-infected (Fig 1A). However, because of the limited sample size at each site, these values should not be interpreted as reflecting differences in infection prevalence between locations.

### Age impact on *Plasmodium* prevalence and multi-species infections consequences on parasitemia in chimpanzees

We analyzed the influence of several host factors, such as age, sex, body temperature, and blood group, on the *Plasmodium* infection status and parasitemia levels in chimpanzees. While body temperature, sex, and blood group showed no significant differences between infected and non-infected animals, age emerged as a key factor associated with infection (Table B in S1 File). Infected chimpanzees were significantly younger than non-infected ones (median age: 118 months (9.8 years) vs. 380 months (31.7 years); Welch’s t-test *P*-value < 0.05; Fig 1B; Table B in S1 File). This pattern was particularly pronounced in animals infected with *P. gaboni* and *P. reichenowi* (PG/PR), and with all three species (*P. gaboni*, *P. reichenowi*, and *P. vivax*-like; PG/PR/PV), who were significantly younger than non-infected chimpanzees (Fig 1B). Analysis of parasitemia levels by qPCR further revealed that multi-species infections were associated with higher parasite burdens. Parasitemia ranged from as low as 5.11 copies/μL in a mono-infected *P. ovale*-like animal to a median of 7.79 copies/μL in bi-infected chimpanzees (PG/PR) and a substantially higher median of 1323.90 copies/μL in tri-infected animals (PG/PR/PV) (Fig 1C). No significant correlation was detected between age and parasitemia levels (Fig 1D). However, interpretation of this relationship is limited because the study population included only two individuals younger than seven years of age, only one of whom was infected. To verify infection status across the entire cohort, qPCR was performed for all individuals, including those classified as nanopore-negative by sequencing. Corresponding Ct values and estimated parasite densities are reported in Table C in S1 File.

### Blood and immunological parameters associated with *Plasmodium* infection status

**Fig 2:**
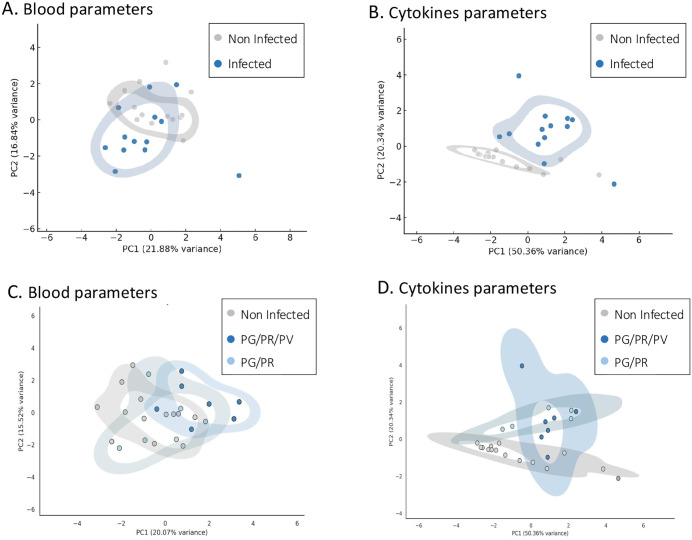
Principal Component Analysis (PCA) of blood and cytokine parameters by *Plasmodium* infection status and species in chimpanzees. **(A)** PCA of 15 hematological and biochemical parameters comparing non-infected and *Plasmodium*-infected chimpanzees. **(B)** PCA of 8 cytokine and chemokine parameters comparing non-infected and infected chimpanzees. **(C)** PCA of hematological and biochemical parameters stratified by infection group: non-infected, PG/PR-infected (*P. gaboni*, *P. reichenowi*), and PG/PR/PV-infected (*P. gaboni*, *P. reichenowi*, *P. vivax*-like). **D.** PCA of cytokine and chemokine parameters stratified by the same infection groups. In all panels **(A–D)**, prediction ellipses represent each group at an 80% confidence level, illustrating the separation between infection categories.

To assess global variation in hematological, biochemical, and immune responses to *Plasmodium* infection, a principal component analysis (PCA) was performed on 15 hematological and 8 cytokine and chemokine parameters. When comparing all infected chimpanzees (PG/PR/PV, PG/PR, and PO) to non-infected animals, blood parameters PCA showed partial separation along PC1 and PC2 (21.88% and 16.84% variance, respectively; Fig 2A). In contrast, cytokine/chemokine-based PCA showed a more pronounced discrimination (PC1 = 50.36%, PC2 = 20.34%; Fig 2B). Stratification by infection group (PG/PR, PG/PR/PV, PO) revealed only weak species-specific patterns. Because the *P. ovale-like* infection was detected in only a single individual (n = 1), this case was not included in statistical comparisons and is shown only for descriptive purposes. While blood parameters PCA did not show clear clustering between non-infected, PG/PR-, and PG/PR/PV-infected animals (Fig 2C), cytokine/ chemokine parameters PCA revealed some separation of PG/PR infected animals from non-infected ones along PC2 (20.34% variance; Fig 2D). Overall, the hematological and biochemical values measured in both infected and non-infected chimpanzees were consistent with previously reported physiological ranges for healthy captive *Pan troglodytes* populations [36]. For example, red blood cell counts in our dataset (mean ≈ 4.5–5.0 × 10⁶/mm³) fall within the reference interval of approximately 3.5–6.0 × 10⁶/mm³, hemoglobin values (≈12–13 g/dL) within the reported 10–16 g/dL, and white blood cell counts (≈9–10 × 10³/mm³) within the typical 4–12 × 10³/mm³ range for the species [36].

**Fig 3:**
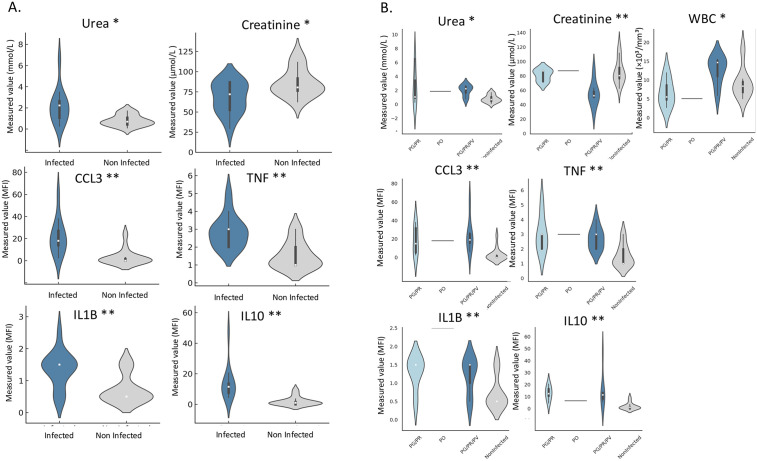
Violin plots of hematological, biochemical and chemokine/cytokine parameters associated significantly with *Plasmodium* infection status in chimpanzees. Each violin plot displays the distribution density for each infection group, with embedded boxplots showing the interquartile range (IQR), central white dots indicating the median, and vertical lines representing the data range (excluding outliers). **(A)** Distribution of the six parameters that differed significantly between *Plasmodium*-infected and non-infected chimpanzees: urea, creatinine, CCL3, TNF, IL-1β, and IL-10. **(B)** Distribution of these same parameters across infection types: PG/PR (n = 5), PG/PR/PV (n = 7), PO (n = 1), and non-infected animals (n = 14). White blood cell count (WBC) and IFN-γ, which were significantly associated with infection status only when stratified by species group, are also shown. Urea levels (mmol/L) reflect nitrogen waste excretion efficiency; creatinine (μmol/L) serves as a marker of kidney function; WBC counts are expressed in ×10^3^/mm^3^. Cytokine and chemokine levels (IL-10, TNF, IL-1β, CCL3 and IFN-γ) correspond exclusively to circulating plasma measurements quantified using a Luminex multiplex assay (ProcartaPlex, Thermo Fisher Scientific) and are reported as median fluorescence intensity (MFI)values. Overall, infected chimpanzees—particularly those with multi-species infections—exhibited elevated urea and inflammatory cytokine levels, along with reduced creatinine and WBC counts. Values are normalized residuals; negatives indicate below-average levels. *Asterisks indicate statistically significant differences between groups based on the linear models described in the Methods section (*P < 0.05; *P < 0.01).

Univariate analyses identified six parameters significantly associated with infection. Infected chimpanzees had higher urea (P-value = 0.012; mean 2.17 mmol/L) and lower creatinine (P-value = 0.014; mean = 67.63 μmol/L) compared to non-infected chimpanzees (mean = 0.80 mmol/L and 86.11 μmol/L, respectively) (Table 1 and Figs 3A and S1). In addition, the urea-to-creatinine ratio was higher in infected chimpanzees (ratio = 0.03) compared to non-infected individuals (ratio = 0.009). Cytokine/chemokine markers IL-10 (P-value = 0.002), CCL3 (P-value = 0.001), TNF (P-value = 0.001), and IL-1β (P-value = 0.003) were also elevated in infected animals (Table 2 and Figs 3B and S2). Upon stratification by infection group (PG/PR, PG/PR/PV), the same six parameters remained significant, and in addition white blood cells (WBC) count also became significantly associated with infection status (P-value = 0.045), with lower counts in PG/PR infected chimpanzees but higher in PG/PR/PV infected chimpanzees (Table 2 and S3 Fig) and IFN-γ became significantly associated with infection status with lower levels in infected individuals (P-value = 0.019; Table 2 and S4 Fig).

**Table 1:** Mean response values and *P*-values for 15 blood parameters and 8 cytokine/chemokine markers in *Plasmodium*-infected and non-infected chimpanzees. *P*-values were generated from linear models comparing both groups. Statistically significant differences (*P* < 0.05) are marked with an asterisk. White blood cell, platelet, lymphocyte, and monocyte counts are expressed in ×10^3^/mm^3^; red blood cell counts in ×10⁶/mm^3^; hemoglobin in g/dL; hematocrit in %; and neutrophils in ×10⁹/L. GGT, ASAT, and ALAT are expressed in U/L; creatinine in μmol/L; and urea, triglycerides, and cholesterol in mmol/L. Cytokine and chemokine levels correspond to circulating plasma measurements quantified using a Luminex multiplex assay (ProcartaPlex, Thermo Fisher Scientific) and are reported as background-adjusted median fluorescence intensity (MFI) values. Statistical significance is indicated by asterisks (*P < 0.05; **P < 0.01).

Parameter	Mean Non Infected	Mean Infected	P-value
White blood cells	9.29	9.78	0.773
Red blood cells	4.53	4.98	0.052
Platelets	253.79	268.54	0.688
Neutrophyls	6.12	6.31	0.908
Monocytes	0.48	0.49	0.914
Lymphocytes	2.21	2.49	0.303
Hemoglobine	12.81	13.15	0.554
Hematocryte	36.52	38.72	0.173
GGT	19.20	13.67	0.148
ASAT	19.04	26.09	0.145
ALAT	16.98	19.07	0.520
Urea*	0.80	2.17	0.012
Creatinine*	86.11	67.63	0.014
Triglycerides	1.20	1.32	0.678
Cholesterol	4.03	3.76	0.368
IL4	7.18	7.58	0.495
CCL3**	3.00	21.42	0.001
CCL5	3223.64	4613.58	0.065
TNF**	1.54	2.85	0.001
IL6	1.57	2.62	0.237
IFNG	1.36	1.23	0.599
IL1B**	0.71	1.35	0.003
IL10**	1.89	13.73	0.002

**Table 2:** Mean response values and *P*-values for 15 blood parameters and 8 cytokine/chemokine markers in non-infected chimpanzees and those infected with different *Plasmodium* species. *Plasmodium* species combinations: PG/PR/PV (n = 7) and PG/PR (n = 5) compared to non-infected individuals (n = 14). *P*-values were obtained from linear models comparing each infected group to the non-infected group. Statistically significant differences (*P* < 0.05) are indicated with an asterisk. White blood cell, platelet, lymphocyte, and monocyte counts are expressed in ×10³/mm³; red blood cell counts in ×10⁶/mm³; hemoglobin in g/dL; hematocrit in %; and neutrophils in ×10⁹/L. GGT, ASAT, and ALAT values are in U/L; creatinine in μmol/L; and urea, triglycerides, and cholesterol in mmol/L. Cytokine and chemokine responses correspond to circulating plasma measurements quantified using a Luminex multiplex assay (ProcartaPlex, Thermo Fisher Scientific) and are reported as background-adjusted median fluorescence intensity (MFI) values. Statistical significance is indicated by asterisks (*P < 0.05; **P < 0.01).

Parameter	Mean Non Infected	Mean PG/PR	Mean PG/PR/PV	P-value
White blood cells*	9.29	6.72	12.64	0.045
Red blood cells	4.53	4.75	5.09	0.120
Platelets	253.79	233.40	309.57	0.2967
Neutrophyls	6.12	3.23	8.88	0.056
Monocytes	0.48	0.46	0.56	0.783
Lymphocytes	2.21	2.50	2.68	0.259
Hemoglobine	12.81	12.44	13.41	0.503
Hematocryte	36.52	37.00	39.01	0.396
GGT	19.20	14.4	12.41	0.323
ASAT	19.04	22.84	22.31	0.554
ALAT	16.98	14.22	18.59	0.440
Urea*	0.80	2.46	2.00	0.025
Creatinine**	86.11	81.04	55.28	0.001
Triglycerides	1.20	1.79	0.98	0.117
Cholesterol	4.03	3.72	3.67	0.549
IL4	7.18	7.00	7.50	0.804
CCL3**	3.00	18.50	24.00	0.003
CCL5	3223.64	3356.70	5098.00	0.003
TNF*	1.54	3.00	2.71	0.042
IL6	1.57	3.20	1.86	0.382
IFNG	1.36	1.00	1.14	0.373
IL1B**	0.71	1.30	1.21	0.003
IL10**	1.89	12.90	15.36	0.003

**Fig 4:**
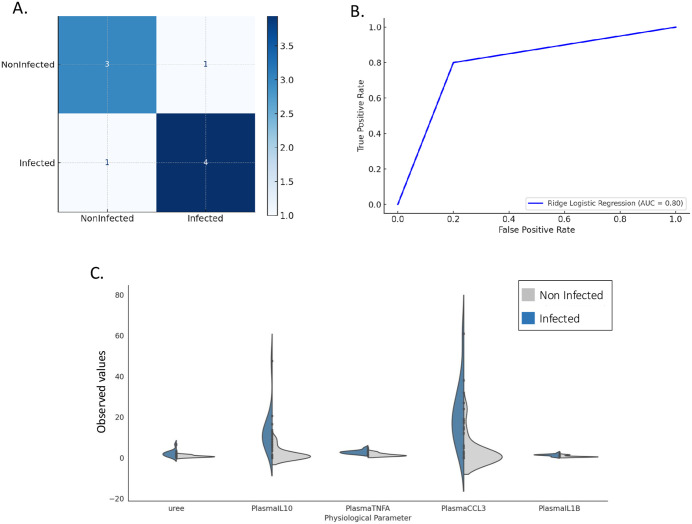
Ridge logistic regression model for predicting *Plasmodium* infection in chimpanzees. **(A)** Confusion matrix from the ridge logistic regression model predicting infection status based on selected physiological and cytokine parameters. The model correctly classified 3 of 4 non-infected and 4 of 5 infected animals, with one misclassification in each group, indicating balanced predictive performance. **(B)** Receiver Operating Characteristic (ROC) curve for the same model, based on principal components derived from physiological and cytokine data. The area under the curve (AUC) is 0.80, reflecting good discriminatory ability. **(C)** Violin plots showing the five parameters retained as significant predictors by LASSO regression: urea, IL-10, TNF, CCL3, and IL-1β. Urea values are expressed in mmol/L; cytokine levels (IL-10, TNF, CCL3, IL-1β) correspond to circulating plasma measurements quantified using a Luminex multiplex assay (ProcartaPlex, Thermo Fisher Scientific) and are reported as median fluorescence intensity (MFI) values. The Y-axis represents the measured values of each parameter in their respective units. Each violin represents the distribution of values by infection status, with internal boxplots indicating the interquartile range (IQR), central white dots showing the median, and vertical lines representing the data range (excluding outliers). These five parameters were consistently identified as the most informative for distinguishing infected from non-infected chimpanzees.

To test whether physiological biomarkers and cytokines predict *Plasmodium* infection status, and to quantify their joint contribution, a ridge-penalized logistic regression was fitted. The model showed balanced discrimination (AUC = 0.80), correctly classifying most infected and non-infected individuals from the combined biomarker and cytokine profiles (Fig 4A and 4B). Consistently, the LASSO regression retained five of these six parameters as the strongest predictors of infection status: urea (coefficient = 0.41), IL-10 (0.53), TNF (0.49), CCL3 (0.38), and IL-1β (0.33) (Fig 4C). All parameters were positively associated with infection status. Although creatinine and WBC were not retained in the penalized model, their significance in univariate and species-stratified analyses suggest broader physiological relevance in the host response to *Plasmodium* infection.

### *Ex vivo* cytokine responses in infected chimpanzees are predominantly anti-inflammatory

Upon *ex vivo* stimulation with PMA or ionomycin directly performed after isolation in the field laboratory, PBMCs isolated from infected chimpanzees produced significantly higher levels of the anti-inflammatory cytokine IL-10 compared to non-infected controls (P-value < 0.05 in both cases; Table 3). This trend remained consistent when analyzing different *Plasmodium* species independently (Table 4).

**Table 3:** Mean response values and *P*-values for eight cytokine parameters measured in PBMC culture supernatants following in vitro stimulation with PMA or ionomycin in *Plasmodium*-infected and non-infected chimpanzees. Cytokine levels were quantified using a Luminex multiplex assay (ProcartaPlex, Thermo Fisher Scientific) and are reported as median fluorescence intensity (MFI) values. P-values were derived from linear models comparing cytokine levels between infected and non-infected groups. Statistically significant differences (P < 0.05) are indicated in bold and marked with an asterisk.

		Mean Non Infected	Mean Infected	P-value
Ionomicine stimulation	IFNG	2.38	19.50	0.967
	IL1B	2.09	13.50	0.843
	IL10*	1.98	81.50	0.003
	IL4	7.69	57.00	0.491
	CCL3	1580.92	10611.50	0.947
	CCL5	2968.15	18744.50	0.946
	TNF	40.69	238.00	0.787
	IL6	32.08	160.50	0.910
PMA stimulation	IFNG	0.85	13.00	0.259
	IL1B	0.87	6.50	0.521
	IL10*	0.13	44.50	0.0007
	IL4	6.31	53.00	0.164
	CCL3	36.73	274.00	0.686
	CCL5	2266.10	16475.00	0.922
	TNF	4.23	41.00	0.164
	IL6	5.00	55.00	0.278

**Table 4:** Mean response values and *P*-values for eight cytokine parameters measured in PBMC culture supernatants following in vitro stimulation with PMA or ionomycin in non-infected chimpanzees and those infected with different *Plasmodium* species combinations. Cytokine responses were quantified using a Luminex multiplex assay (ProcartaPlex, Thermo Fisher Scientific) and are reported as median fluorescence intensity (MFI) values. P-values were calculated using linear models comparing each infected group to the non-infected group. Statistically significant differences (P < 0.05) are shown in bold and marked with an asterisk.

		Mean Non Infected	Mean PG/PR	Mean PG/PR/PV	P-value
Ionomicine stimulation	IFNG	2.38	2.67	2.67	0.967
	IL1B	2.09	1.83	1.83	0.843
	IL10*	1.98	17.83	17.83	0.003
	IL4	7.69	8.00	8.00	0.491
	CCL3	1580.92	1686.80	1686.80	0.947
	CCL5	2968.15	2514.70	2514.70	0.946
	TNF	40.69	37.67	37.67	0.787
	IL6	32.08	10.67	46.25	0.910
PMA stimulation	IFNG	0.85	1.67	1.50	0.259
	IL1B	0.87	0.83	2.00	0.521
	IL10*	0.13	11.17	6.00	0.0007
	IL4	6.31	7.33	7.50	0.164
	CCL3	36.73	28.67	85.00	0.686
	CCL5	2266.10	2615.5	2970.80	0.9222
	TNF	4.23	5.67	8.00	0.164
	IL6	5.00	5.68	6.00	0.278

## Discussion

Although the health effects of *P. falciparum* and *P. vivax* infections in humans are well characterized, including fever, anemia, and systemic inflammation in symptomatic cases, the physiological consequences of *Plasmodium* infections in great apes remain largely unknown. To address this, we assessed hematological, biochemical, and immunological markers in 27 semi-wild chimpanzees in Gabon through field-based sample processing. The results showed that *Plasmodium* infection was highly prevalent (48.15%) among chimpanzees, with infected individuals being significantly younger than non-infected ones. In addition, chimpanzees with multi-species infections, particularly PG/PR/PV tri-infections, exhibited higher parasitemia levels, suggesting possible additive effects of co-infection (Fig 1) [37–40]. Importantly, most hematological and biochemical values measured in both infected and non-infected chimpanzees fell within the physiological ranges previously reported for healthy captive *P. troglodytes* populations [36]. Although several parameters differed statistically between infection groups, their absolute values remained within expected biological limits for the species. This pattern suggests that the infections observed here are unlikely to be associated with overt clinical pathology. Because behavioral symptoms cannot be reliably assessed in sedated chimpanzees during routine veterinary procedures, our interpretation relies primarily on objective physiological measurements, including parasite densities, hematological parameters, biochemical markers, cytokine profiles, and body temperature. Body temperature was measured at the time of sampling, and no significant differences were detected between infected and non-infected individuals, indicating the absence of detectable fever at the time of sampling. Nevertheless, infected chimpanzees exhibited altered urea and creatinine levels, species-specific changes in white blood cell counts, and elevated concentrations of certain pro- and anti-inflammatory cytokines, indicating systemic immune activation and possible long-term immunomodulation.

Regarding age patterns and infection burden, infected chimpanzees in our cohort were younger than non-infected individuals, although no significant correlation was detected between age and parasitemia (Fig 1B and 1D) [32,41–44]. Interpretation of these patterns nevertheless remains limited by the age structure of the sampled population, which included only two individuals younger than seven years old. In humans living in endemic areas, the highest burden of malaria infection and disease occurs in infants and school-aged children before the gradual acquisition of partial immunity through repeated exposure. Whether similar age-dependent infection dynamics occur in African great apes remains unresolved and will require larger longitudinal studies including more juvenile individuals. Nevertheless, similar age-related declines in *Plasmodium* infection prevalence have been reported in wild chimpanzees and mandrills, suggesting that repeated exposure may contribute to the development of partial immune control in African primates [15,45].

Regarding hematological, biochemical, and immunological consequences of chronic *Plasmodium* infection in chimpanzees, 23 key markers were compared between infected and uninfected animals, revealing distinct patterns with important implications for disease tolerance and host-pathogen dynamics.

Biochemical analysis revealed that infected animals had an elevated urea-to-creatinine ratio, indicative of pre-renal dysfunction commonly associated with impaired renal hypoperfusion in humans. Despite the statistical increase in urea levels in infected individuals, mean values remained within the physiological range reported for healthy chimpanzees [36], suggesting that these differences likely reflect subtle metabolic or hydration changes rather than clinically significant renal dysfunction. In the context of chronic malaria, kidney function can be significantly affected, potentially leading to complications such as malarial nephropathy and acute kidney injury (AKI) [46]. These renal impairments may result from a combination of hemodynamic disturbances, immune-mediated damage, and systemic effects of prolonged parasitemia. While AKI is more frequently reported in severe malaria cases, emerging evidence suggests that chronic infections can also lead to subclinical or progressive renal dysfunction [46]. In this study, these biochemical and immunological signatures in asymptomatically infected chimpanzees may therefore reflect early or mild forms of renal stress linked to chronic immune activation and parasite persistence. In humans, symptomatic malaria is typically associated with fever, anemia, and marked inflammatory responses, whereas asymptomatic infections often present with low parasite densities and more moderate physiological alterations [47]. The parasite densities and physiological markers observed in this study are therefore more consistent with patterns reported in low-grade or asymptomatic malaria infections in humans.

Regarding immune responses, plasma cytokine measurements revealed that infected chimpanzees exhibited significantly elevated concentrations of TNF and IL-10 compared to non-infected animals, consistent with reports in humans with asymptomatic *P. falciparum* infections [5]. TNF, a pro-inflammatory cytokine produced primarily by γδ T cells and CD14 ⁺ monocytes during malaria infection in humans, plays a key role in controlling parasitemia, but can cause damage if not tightly regulated [48–50]. Conversely, IL-10 (anti-inflammatory), primarily produced by IFN-γ ⁺ Th1 cells and regulatory T cells in response to chronic antigenic stimulation, counteracts inflammation and limits immunopathology, a phenomenon frequently observed in asymptomatic falciparum malaria [51–54]. The elevated IL-10 responses observed in infected chimpanzees are consistent with a regulatory immune profile that may contribute to the modulation of inflammatory responses during repeated or chronic exposure to *Plasmodium* parasites. This is consistent with observations in humans living in high-transmission settings, where IL-10 produced by IFN-γ–producing CD4 ⁺ Th1 cells helps control both immunopathology and clinical symptoms [53,54]. Together, these results suggest the existence of a finely regulated balance between pro-inflammatory (TNF) and anti-inflammatory (IL-10) responses in enabling infected chimpanzees to tolerate persistent *Plasmodium* infection while minimizing clinical disease. Similar cytokine patterns characterized by balanced pro- and anti-inflammatory responses have been reported in humans with asymptomatic or chronically exposed malaria infections, where regulatory responses such as IL-10 production help limit immunopathology despite persistent parasitemia [55].

**Fig 5:**
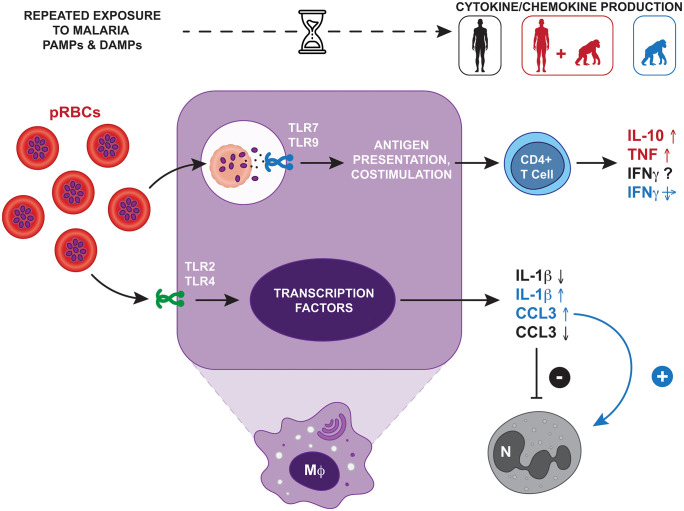
Comparative model of cytokine and chemokine responses in humans and chimpanzees with chronic *Plasmodium* infection. With repeated exposure to *Plasmodium*-parasitised red blood cells (pRBCs), both humans and chimpanzees develop immune tolerance, leading to asymptomatic infections. In humans, an initial pro-inflammatory response (e.g., TNF, IL-1β, CCL3) shifts over time toward a more regulated profile, marked by increased IL-10 production by CD4 ⁺ T cells and decreased IL-1β and CCL3, leading to an inactivation of neutrophils over time (-). In contrast, IL-1β and CCL3 remain elevated in chronically infected chimpanzees, suggesting sustained immune activation involving neutrophils (+). Effector mechanisms such as phagocytosis, phagolysosome activation, antigen presentation, T cell co-stimulation, and IFN-γ production by CD4 ⁺ T cells are enhanced in humans through Toll-like receptor (TLR) signaling, which recognizes *Plasmodium* pathogen-associated molecular patterns (PAMPs) and damage-associated molecular patterns (DAMPs) (PMID: 25324127). These mechanisms help maintain parasite control without clinical symptoms. The role of IFN-γ in this regulatory sequence remains debated in humans but similar levels are seen in both healthy and infected chimpanzees and IFN concentrations decrease in bi- and tri-infected animals compared to controls. Cytokine and chemokine profiles observed in our animal cohort are compared to known responses in humans: **black** = human-specific responses; **red** = shared trends in humans and chimpanzees; **blue** = responses observed only in chimpanzees (adapted from PMID: 24743880). Mφ = macrophage; N = neutrophil. Human and chimpanzee icons were drawn by the authors.

Beyond TNF and IL-10, plasma measurements also showed a significant increase in CCL3 and IL-1β levels among infected chimpanzees. CCL3, a chemokine typically downregulated in asymptomatic malaria in humans [55], has been associated with the development of severe disease, including cerebral and placental malaria [56,57]. Similarly, IL-1β is a hallmark of acute malaria infection and a marker of disease severity in humans [58]. The concurrent upregulation of CCL3 and IL-1β observed in infected chimpanzees may reflect species-specific differences in immune regulation, including leukocyte recruitment (notably neutrophils), inflammatory response thresholds, or parasite clearance mechanisms. These differences could also involve differences in erythrocyte sequestration [59] or parasite-driven immune evasion strategies [4] (Fig 5). The concurrent increase in CCL3 and IL-1β underscores the need to further investigate whether these immune responses confer enhanced parasite control, increased risk of immunopathology, or represent a unique adaptation of chimpanzees to chronic malaria exposure.

When comparing chimpanzees with bi- and tri-species *Plasmodium* infections to non-infected ones, similar profiles emerged. Tri-infected animals (*P. gaboni*, *P. reichenowi*, and *P. vivax*-like) displayed higher white blood cell counts, higher urea and lower creatinine levels, and a cytokine profile marked by increased TNF, CCL3, IL-1β, and IL-10, relative to bi-infected animals (*P. gaboni* and *P. reichenowi*). Notably, IFN-γ levels were significantly lower in multiple infections, suggesting a dampened Th1 response. These patterns suggest that co-infection with *P. vivax*-like may alter immune regulation, potentially shifting the host response toward a more anti-inflammatory or immunomodulatory profile, possibly through enhanced IL-10-mediated suppression of pro-inflammatory pathways combined with reduced IFN-γ activity. This may reflect immune exhaustion, tolerance, or parasite-driven immune evasion mechanisms in the context of chronic, multi-species malaria infections. Overall, these results highlight the possible role of *P. vivax-like* co-infection in further modulating immune responses, possibly enhancing immune tolerance and facilitating persistent, asymptomatic multi-species malaria infection in chimpanzees.

PBMC stimulation assays showed that PBMCs from infected chimpanzees exhibited significantly higher IL-10 upon stimulation with ionomycin or PMA compared to non-infected controls, while pro-inflammatory cytokine production (TNF, IFN-γ, IL-6) remained low. This result further supports our earlier observations of elevated systemic IL-10 levels in infected animals, underscoring the prominence of an immunoregulatory state in response to chronic *Plasmodium* infection. Such a profile is consistent with reports from *P. falciparum*-endemic regions, where repeated infections dampen monocyte activation and inflammatory responses [60,61]. Such IL-10–dominated profiles may reflect immune tolerance or epigenetic reprogramming of monocytes, as seen in asymptomatic malaria cases in Malian adults and children, potentially increasing susceptibility to secondary infections [55]. Together, these findings highlight the central role of IL-10 in orchestrating both systemic and cellular immune regulation during chronic malaria exposure in chimpanzees. Serological data on past exposures would help clarify the role of chronic infection in shaping this regulatory phenotype.

Overall, this study advances our understanding of the hematological and immune landscape in wild chimpanzees with asymptomatic *Plasmodium* infections, revealing striking similarities to the immune responses observed in human malaria. Contrary to the notion that chronic malaria is immunologically silent, our results suggest that infected chimpanzees undergo significant shifts in cytokine profiles, particularly elevated systemic and cellular IL-10 levels. This pattern suggests a state of immunosuppressive priming, which may enable the host to tolerate persistent parasitemia, but could also compromise defenses against other pathogens. In addition, the detection of subtle alterations in renal function, especially in chimpanzees with tri-species infections, further indicates that even asymptomatic malaria can exert physiological impacts.

Although infected individuals did not show detectable physiological disturbances in the parameters measured in this study, disease expression may vary depending on the host and parasite species. Clinical cases of *P. reichenowi* in chimpanzees [20], and of *Plasmodium pitheci* in rehabilitated Bornean orangutans have been documented [19]. Persistent immune stimulation may lead to immune exhaustion or paralysis, potentially increasing susceptibility to other infections. This highlights the importance of ongoing immune monitoring, particularly in juveniles with multi-species infections. Accordingly, management plans should consider immune health alongside population size, as asymptomatic infections can carry hidden costs. Field-feasible panels, complete blood counts, and simple immune assays (e.g., inflammatory markers or parasite-specific antibodies) could serve as indicators of population health, especially in juveniles with multi-species infections.

Finally, several limitations in this study should be acknowledged, particularly the modest sample size, restriction to asymptomatic individuals, and a limited biomarkers panel. To overcome such limit, it would be interesting in the future to expand immune profiling to include additional regulatory markers (e.g., IL-12, TGF-β) and implementing longitudinal and serological monitoring for a more comprehensive understanding of immune memory, reinfection, and long-term health outcomes in these endangered populations.

In summary, this study suggests that ostensibly asymptomatic malaria in chimpanzees is not benign: immune modulation varies with infection complexity and age, a pattern compatible with virulence expressed as subclinical costs (e.g., anemia risk, altered inflammatory tone, reduced condition). Together with accumulating reports in other primates that malaria affects physiology and performance even without overt illness [62], this argues for integrating longitudinal health surveillance into conservation practice.

## Methods

### Ethics statement

All animal procedures were conducted in strict accordance with national and international guidelines for animal welfare to ensure the well-being of the animals. Veterinary staff from La Lékédi Park and the Centre Interdisciplinaire de Recherches Médicales de Franceville (CIRMF) supervised all health assessments and blood collection. Ethical approval was obtained from the government of the Republic of Gabon and the Animal Life Administration of Libreville (CITES 00956), as well as the Animal Welfare and Ethical Review Body of the London School of Hygiene & Tropical Medicine (LSHTM; 0020/2013/SG/CNE).

### Study sites and sample collection

Blood samples were collected from 27 chimpanzees as part of routine veterinary health assessments at two sites in Gabon: La Lékédi Park (N = 9) and the Primatology Center at CIRMF, Franceville (N = 18) (Fig 1A). Sampling took place during peak malaria transmission seasons—October 2018 at La Lékédi (5 females, 4 males) and May 2019 at CIRMF (11 females, 11 males). Four animals were excluded due to incomplete data, yielding a final sample size of 27. During assessments, chimpanzees were anesthetized with an intramuscular combination of medetomidine (0.03–0.06 mg/kg IM) and ketamine (2–6 mg/kg IM; Imalgene) delivered via blowpipe; for adults, ketamine was typically 3 mg/kg within this range, with medetomidine adjusted according to clinical judgement. Blood was collected from the iliac vein within 15 minutes of sedation to minimize stress-related alterations in hematological parameters. Three blood samples were obtained from each animal using dry, EDTA, and heparin tubes for subsequent analysis. Age, weight, and rectal temperature were also recorded.

### *Plasmodium* screening, parasitemia quantification and species identification

To reduce host DNA within samples, 4 mL of EDTA-collected chimpanzee blood was diluted with PBS and passed through CF11 cellulose columns for leukocyte depletion [63]. Genomic DNA was extracted using the DNeasy Blood and Tissue Kit (Qiagen, France) following the manufacturer’s instructions. *Plasmodium* species were identified by nested PCR amplification and sequencing of the cytochrome *b* (cyt-b) gene using two primer sets: DW2–DW4 [64]. PCR products were visualized via 1.2% agarose gel electrophoresis and quantified with a Qubit fluorometer.

Due to the initial PCR protocol’s limited sensitivity for detecting *P. vivax*-like parasites, all DNA samples were additionally screened using a conventional PCR targeting the mitochondrial cytochrome oxidase I (*cox1*) gene, following the protocol of Liu et al. [34]. Amplified products were resolved on 1.5% agarose gels and sequenced by Eurofins MWG. Resulting sequences were analyzed using BLAST for species identification (https://blast.ncbi.nlm.nih.gov/Blast.cgi).

Parasite load was then quantified using a quantitative PCR (qPCR) assay targeting the *Plasmodium* cytochrome *b* gene, allowing amplification of multiple *Plasmodium* species including *P. vivax-like* parasites. The assay used the primers ParaF (5′-CTATGCTTTATTATGGATTGGATG-3′) and ParaR (5′-GAGCTGTAATCATAATGTGTTC-3′). qPCR reactions (20 μL total volume) contained 1 μL of DNA template, 10 μM of each primer, and 1 × EvaGreen master mix. Amplification was performed on a LightCycler 96 system with the following cycling conditions: 95 °C for 10 min; 40 cycles of 95 °C for 15 s, 60 °C for 20 s, and 72 °C for 20 s; followed by 95 °C for 10 s and 55 °C for 1 min. Standards were run in duplicate across a dilution range from 300,000–1 parasite/μL. Sample parasitemia was estimated by extrapolating Ct values against the standard curve using LightCycler 96 software.

Parasitemia estimates were calibrated using parasite densities determined from Giemsa-stained thin blood smears. Parasite counts were obtained across eight distant microscopic fields, each containing approximately 200 red blood cells (RBCs), and parasite density was calculated using the formula:


$$\begin{array}{c} Parasites/L\;=\;\left(\frac{Parasitized\;RBCs}{Total\;RBCs}\right)\;\times \;\text{4}.\text{5}\;\times \;{\text{1}\text{0}}^{\text{6}} \end{array}$$

This formula corresponds to the standard conversion from percentage parasitemia on thin blood films to parasites per volume of blood. Parasite density was expressed as parasites per µL using an assumed RBC count of 4.5 × 10⁶ RBCs/µL when individual complete blood count (CBC) values were unavailable (CDC DPDx; BSH guideline).

Only samples that were positive by both nested PCR and qPCR were subsequently selected for high-throughput sequencing. This selection strategy was implemented to optimize sequencing resources by focusing nanopore sequencing on samples with confirmed parasite DNA.

Sequencing was performed using high-throughput technology (MinION) to allow the identification of mixed-infections. DNA libraries were prepared using the Ligation Sequencing Kit (SQK-NBD114.96) and Native Barcoding Kit 96 V4, then sequenced on a MinION Mk1C device (Oxford Nanopore Technologies) with FLO-MIN114 (R10.4.1) flow cells, targeting 100,000–200,000 reads per sample. For species identification, a standardized random subsample of 10,000 reads per chimpanzee was BLASTed against a custom Cytb database. This sub-sampling approach optimized computational efficiency and ensured cross-sample comparability without compromising species-level resolution.

### Chimpanzees blood group status

Given the known association between ABO blood group and malaria susceptibility in humans [65], ABO blood group and rhesus (Rh) status were determined for each chimpanzee using the SERAFOL ABO + D kit within 3 hours of blood collection. Blood samples were tested for agglutination with specific antisera to identify blood group and Rh status.

### Blood numeration and serum biochemical analyses

To evaluate the impact of *Plasmodium* infection on chimpanzee health, we assessed eight hematological parameters commonly altered in human malaria, including total white blood cell (WBC), red blood cell (RBC), platelet, neutrophil, monocyte, and lymphocyte counts, as well as hemoglobin and hematocrit levels, using an automated hematology analyzer (Hematology Analyser ACT 10, Beckman Coulter, USA). Complete blood counts were performed within 90 minutes of sample collection at the CIRMF Medical Analysis Center. Renal (urea, plasma creatinine) and hepatic (ALAT, ASAT, GGT) function markers were also measured, given the known impact of malaria on these organs in humans, using a fully automated clinical chemistry analyzer (Hitachi Model 902 Automatic Analyser, Roche Diagnostics, France), following the manufacturer’s protocols. In addition, total cholesterol and triglyceride levels were analyzed, given the auxotrophic dependence of *Plasmodium* parasites on host cholesterol. Overall, the hematological and biochemical parameters measured in this study fell within the physiological ranges previously reported for healthy chimpanzees [36]. Although several parameters differed statistically between infected and non-infected individuals, their absolute values generally remained within the expected biological limits for the species.

### Peripheral blood mononuclear cell (PBMC) stimulation

To assess immune status, we used phorbol myristate acetate (PMA) and ionomycin stimulation, a widely established method for inducing immune responses in vitro across multiple species, including humans [66]. This approach triggers rapid secretion of proinflammatory cytokines and chemokines within six hours of stimulation [67]. For each animal, 4 mL of heparinized whole blood was processed within 3 hours of collection. Samples were centrifuged (800 × g, 20 min), and plasma was aliquoted and stored at –80 °C. The buffy coat, containing peripheral blood mononuclear cells (PBMCs), was transferred to 24-well plates and stimulated with PMA (25 ng/mL) and ionomycin (1 μg/mL) for 6 hours. After incubation, the cells were centrifuged, and the supernatants were collected and stored at –80 °C for subsequent analysis of cytokines and chemokines.

### Cytokine and chemokine quantification by multiplex Luminex assay

To investigate immune responses associated with malaria infections, we selected eight cytokines and chemokines previously implicated in malaria-associated inflammation and immune regulation in humans (TNF, IL-6, IFN-γ, IL-1β, IL-4, CCL3/MIP-1α, CCL5/RANTES, and IL-10). Cytokine and chemokine levels were measured using the ProcartaPlex Non-Human Primate Cytokine & Chemokine Panel 30-plex (Thermo Fisher Scientific/ Invitrogen; catalogue EPX300-40044-901) following the manufacturer’s instructions. For each chimpanzee, three sample types were analyzed on the same assay plate: one circulating plasma sample representing baseline cytokine levels, one PMA-stimulated PBMC supernatant, and one ionomycin-stimulated PBMC supernatant. PBMCs were cultured in complete RPMI medium in 96-well plates and stimulated with PMA or ionomycin. For stimulation assays, 1 × 10^5^ PBMCs were cultured per well in 200 μL of complete RPMI medium. After incubation, culture supernatants were collected and stored at −80 °C until analysis. All samples were run in single replicate. Assays were performed in a total reaction volume of 50 μL reaction volume. Plasma samples were analyzed using 25 μL of undiluted plasma, while 50 μL of undiluted PBMC supernatant was used for stimulated samples, in accordance with the manufacturer’s recommended protocol. Median fluorescence intensity (MFI) values were acquired using a MAGPIX Luminex platform (Luminex Corp., Austin, USA) and processed with xPONENT 4.2 software. Cytokine responses were analyzed using background-adjusted MFI values rather than interpolated concentrations, because several analytes fell below the lower limit of quantification of the standard curves. Using MFI values preserves signal resolution among low-responding samples and is commonly applied in malaria immunology and sero-epidemiology studies when reliable concentration estimates cannot be derived from standard curves [68,69]. All statistical analyses were therefore performed on background-adjusted MFI values, and results are interpreted as relative differences in cytokine abundance rather than absolute circulating concentrations.

### Statistical analyses

All statistical analyses have been performed using R version 4.3.1 software [70].

(a) *Data preparation and quality control*

The dataset comprised 15 hematological and 8 cytokine/chemokine parameters measured in chimpanzees sampled in Gabon, along with demographic variables (age, weight, sex, temperature, blood group) and *Plasmodium* infection status. Before analysis, continuous variables were screened for missing values and outliers. For multivariate analyses, hematological, biochemical, and cytokine variables were standardized using z-score transformation (subtracting the mean and dividing by the standard deviation) to ensure that parameters measured on different scales contributed equally to the analyses. Data processing and preparation were performed using the tidyverse R package [71].

(b) *Global comparison of infected vs. non-infected animals*

To assess physiological differences associated with *Plasmodium* infection, all infected animals were compared as a single group to non-infected chimpanzees. Mean values were calculated for each of the 23 physiological parameters (15 hematological parameters, 8 cytokines and chemokines). Prior to statistical testing, the distribution of continuous physiological variables (hematological parameters, biochemical markers, and cytokine measurements) was assessed using Shapiro–Wilk tests (Table D in S1 File) and visual inspection of Q–Q plots. Although some variables showed mild deviations from strict normality due to the relatively small sample size, the distributions were generally symmetrical. Because several variables showed unequal variances between groups, Welch’s t-tests were used to compare group means. This test provides robust control of type I error under heteroscedasticity and moderate deviations from normality [72]. Statistical significance was defined as *P-*value < 0.05. Associations between demographic variables and infection status were thus assessed using Welch’s t-tests for continuous variables (age, weight, temperature) and Fisher’s exact tests for categorical variables (sex, blood group). Because infected chimpanzees were, on average, younger than non-infected individuals, we accounted for potential confounding by including age as a covariate in the regression models used to test associations between infection status and physiological parameters.

Data distributions and group differences were visualized with the ggplot2 package [73] with violin plots displaying kernel density estimates and embedded boxplots (median and IQR), colored by infection status (light grey = non-infected; steel blue = infected). Statistically significant differences were annotated with asterisks (*P*-value < 0.05). To explore global variation in physiological profiles, Principal Component Analysis (PCA) was conducted separately for blood and cytokine parameters using the prcomp() function. Prior to PCA, variables were standardized using z-score transformation as described above. Shaded ellipses represent 60% confidence regions derived from a bivariate normal fit to PC1–PC2 scores for each group. Variables with Pearson correlation coefficients > 0.9 were excluded to reduce multicollinearity [74].

(c) *Subgroup comparison by Plasmodium species combinations*

To investigate whether specific *Plasmodium* species or species combinations differentially influenced physiological responses, infected animals were subdivided into two groups: PG/PR, and PG/PR/PV (PO being alone was not considered). Prior to statistical analyses, the distribution of continuous physiological variables (hematological parameters, biochemical markers, and cytokine measurements) was assessed using Shapiro–Wilk tests and visual inspection of Q–Q plots. Because several variables showed unequal variances and the sample sizes between groups were small, group comparisons were performed using Welch’s t-tests, which are robust to heteroscedasticity and moderate deviations from normality [72]. For each comparison, the direction of change, *P*-value, and statistical significance (*P*-value < 0.05) were recorded. In addition, violin plots were used to visualize all four groups and PO, with distinct colors assigned: PG/PR (light blue), PO (medium blue), PG/PR/PV (dark blue), and non-infected (light grey). To further explore global variation in physiological profiles across infection groups, PCA analyses were conducted on z-score standardized variables, following the same procedure described above. For each variable, values were centered and scaled by subtracting the mean and dividing by the standard deviation, ensuring that parameters measured on different scales contributed equally to the analysis. Shaded ellipses represent 60% confidence regions derived from a bivariate normal fit to PC1–PC2 scores for each group. Variables with Pearson correlation coefficients > 0.9 were excluded prior to PCA to reduce multicollinearity.

(d) *Penalized regression and predictive modeling of infection status*

To identify physiological markers predictive of *Plasmodium* infection (across all species) in chimpanzees, we applied penalized logistic regression using the LASSO (Least Absolute Shrinkage and Selection Operator) method implemented in the glmnet package in R [75]. All predictor variables (hematological parameters, biochemical markers, and cytokine measurements) were standardized to z-scores prior to model fitting by centering each variable to a mean of zero and scaling to a standard deviation of one. This transformation ensures comparability among predictors measured in different units and prevents variables with larger numeric ranges from disproportionately influencing the regression model. Age was included as a covariate to control for potential confounding. The optimal regularization parameter (λ) was selected using 10-fold cross-validation, and variables with non-zero coefficients at λmin were retained as key predictors.

Five variables were identified as most informative: urea, IL-10, TNF, CCL3, and IL-1β. Violin plots were generated using ggplot2 to visualize the distributions of urea and cytokines (IL-10, TNF, CCL3, and IL-1β) by infection status, with urea expressed in mmol/L and cytokines reported as median fluorescence intensity (MFI) from plasma Luminex assays. Model performance was evaluated using a confusion matrix and ROC curve (p_ROC package), correctly classifying 80% infected and 75% non-infected animals, with an AUC of 0.80, indicating go discriminatory power [76].

## Supporting information

S1 File Including: Table A. Summary of BLAST results for *Plasmodium* species detection in chimpanzee samples using MinION sequencing. This table presents the results of a BLAST analysis performed on 10,000 randomly selected reads from MinION sequencing data. Each row corresponds to a different chimpanzee sample, and the columns represent the number of reads matching specific *Plasmodium* species. PCR amplification of the cytochrome B gene was initially used to detect the presence of *Plasmodium* species, followed by MinION sequencing. Only samples that tested positive for *Plasmodium* during the initial PCR and qPCR screening (n = 13 of 27 individuals) were subjected to MinION sequencing, which explains why this table includes results for 13 individuals only. The species identified include *P. adleri*, *P. gaboni*, *P. ovale-like*, *P. praefalciparum*, *P. reichenowi*, *P. vivax-like*, *P. billcollinsi*, *P. malariae-like*, and *P. blacklocki*. The “Non-identified” column indicates the number of reads that could not be confidently assigned to any specific *Plasmodium*species. The final column totals the number of identified reads for each sample. Names followed by an asterisk (*) indicate individuals PCR-positive for *P. vivax-like* based on the *cox1*-specific assay. Table B. Impact of demographic and physiological variables on malaria infection status in chimpanzees in Gabon. This table summarizes the statistical tests used to assess whether host demographic variables were associated with *Plasmodium* infection status (infected vs. non-infected). Continuous variables (age, weight, and body temperature) were compared between groups using Welch’s t-tests, which are robust to unequal variances and sample sizes. Median and interquartile ranges (IQR) are provided to illustrate the distribution of the variables in each group. Categorical variables (sex and blood group) were evaluated using Fisher’s exact tests, which are appropriate for contingency tables with small sample sizes. For each comparison, the test statistic and corresponding P-value are reported. Table C. Concordance between MinION sequencing, species-specific PCR, and qPCR estimates of Plasmodium infection and parasitemia in chimpanzee blood samples from Gabon. This table summarizes the detection of *Plasmodium* parasites in chimpanzee blood samples using three complementary approaches: nanopore sequencing (MinION), species-specific PCR assays, and quantitative PCR (qPCR) estimation of parasite density. For each individual, the table reports the infection status determined from MinION sequencing, the number of sequencing reads assigned to the three main *Laverania* parasites detected in this study (*Plasmodium gaboni*, *P. reichenowi*, and *P. ovale-like*), and the result of a targeted PCR assay designed to detect *P. vivax-like* parasites. MinION sequencing was performed on samples that were positive by nested PCR and qPCR, and species identification was based on BLAST assignment of a standardized random subset of 10,000 cytb reads per sample against a curated *Plasmodium* reference database. Infection status was considered positive when parasite reads were detected within this subsample. For individuals classified as negative by molecular screening, nanopore sequencing was not performed; corresponding read counts are therefore indicated as NA. The column “*P. vivax-like* PCR” indicates the result of a species-specific PCR assay targeting the cytochrome b gene used to detect the presence of *P. vivax-like* parasites. Samples are classified as Positive or Negative according to amplification results. Parasite density was quantified by quantitative PCR targeting the *Plasmodium* cytochrome b gene. The qPCR cycle threshold (Ct/Cq) values are reported for each sample. Parasitemia (parasites per µL of blood) was estimated by comparing sample Ct values to a standard curve generated from serial dilutions of *Plasmodium falciparum* ring-stage parasites of known concentration. The relationship between qPCR concentration estimates and parasite density was calculated using the standard conversion formula: Parasites/µL = (% parasitized red blood cells) × (4.5 × 10⁶ red blood cells per µL), where 4.5 × 10⁶ cells/µL represents the assumed average erythrocyte concentration when individual complete blood counts were unavailable. Very low parasite densities detected in individuals classified as negative correspond to Ct values close to the detection limit of the assay and likely represent background signal or extremely low parasite DNA levels below the threshold for confident infection detection. Ct values and parasitemia values are reported to three decimal places where applicable. “NA” indicates data not available because the corresponding assay was not performed. Table D. Shapiro–Wilk normality tests for all variables measured in chimpanzees. This table presents the results of Shapiro–Wilk tests used to assess the normality of the distribution of each continuous physiological variable prior to statistical comparisons between infected and non-infected chimpanzees. Variables include hematological parameters, biochemical markers, and cytokine/chemokine measurements. W corresponds to the Shapiro–Wilk test statistic and P-values indicate whether the distribution significantly deviates from normality (P < 0.05). Several biochemical and cytokine variables show deviations from strict normality, which is common for immune and metabolic biomarkers. Because sample sizes were small and some variables showed unequal variances between groups, Welch’s t-tests were used for group comparisons as they are robust to heteroscedasticity and moderate deviations from normality. Table E. Complete dataset underlying the hematological, biochemical, parasitological, and immunological analyses performed in this study. The table contains individual-level measurements for all chimpanzees included in the study (n = 27). For each individual, the dataset reports demographic information (name, age in months, weight, sex, and blood group), malaria infection status, parasite density (parasites/µL), and the *Plasmodium* species detected. Body temperature recorded during veterinary examination is also provided. Hematological parameters include white blood cell count (WBC), red blood cell count (RBC), platelet count (PQT), neutrophils (Neut), monocytes (Mono), lymphocytes (Lymph), hemoglobin (Hb), and hematocrit (Ht). White blood cells (WBC), platelets (PQT), lymphocytes (Lymph), and monocytes (Mono) are expressed in ×10³/mm³. Red blood cell count (RBC) is expressed in ×10⁶/mm³. Hemoglobin (Hb) is reported in g/dL and hematocrit (Ht) in percentage (%). Neutrophils (Neut) are expressed in ×10⁹/L. Biochemical parameters include gamma-glutamyl transferase (GGT), aspartate aminotransferase (ASAT), alanine aminotransferase (ALAT), urea, creatinine (creat), triglycerides (TG), and cholesterol (Chol). ALAT, ASAT, and GGT values are expressed in units per liter (U/L). Cholesterol (Chol), triglycerides (TG), and urea concentrations are reported in mmol/L, while creatinine (creat) is expressed in μmol/L. Immunological measurements correspond to circulating cytokine and chemokine levels quantified from plasma using a multiplex Luminex assay (ProcartaPlex Non-Human Primate Cytokine & Chemokine Panel). Reported markers include IL-4, CCL3 (MIP-1α), CCL5 (RANTES), TNF-α, IL-6, IFN-γ, IL-1β, and IL-10. Cytokine responses are reported as background-adjusted median fluorescence intensity (MFI) values.
(DOCX)

S1 Fig Violin plots of blood parameters in *Plasmodium*-infected and non-infected chimpanzees. This figure displays the distribution of 15 blood parameters measured in *Plasmodium*-infected and non-infected chimpanzees. Each violin plot shows the full distribution of values, with an embedded box plot indicating the interquartile range (IQR), the median (central white dot), and the data range (excluding outliers). ALAT, ASAT, and GGT values are expressed in units per liter (U/L). Cholesterol (Chol), triglycerides (TG), and urea concentrations are reported in mmol/L. Creatinine (creat) is expressed in μmol/L. White blood cells (WBC), platelets (PQT), lymphocytes (Lymph), and monocytes (Mono) are presented in ×10³/mm³. Red blood cell count (RBC) is expressed in ×10⁶/mm³. Hemoglobin (Hb) is reported in g/dL, and hematocrit (Ht) in percentage (%). Neutrophils (Neut) are expressed in ×10⁹/L. These plots allow visual comparison of central tendencies and distributional differences in blood markers between infected and non-infected animals, highlighting potential physiological changes associated with *Plasmodium* infection. Values are z-score standardized (centered to mean = 0 and scaled to standard deviation = 1); negative values indicate measurements below the overall mean and positive values indicate above-average levels. Statistical significance is indicated by asterisks (*P < 0.05).
(TIFF)

S2 Fig Violin plots of cytokine and chemokine levels in *Plasmodium*-infected and non-infected chimpanzees. This figure presents the distribution of eight cytokine and chemokine parameters measured in circulating plasma samples from chimpanzees infected with *Plasmodium* and those that were not infected. Cytokine and chemokine levels were quantified using a Luminex multiplex assay (ProcartaPlex Non-Human Primate Cytokine & Chemokine Panel 30-plex, Thermo Fisher Scientific) and are reported as background-adjusted median fluorescence intensity (MFI) values. Each violin plot displays the full distribution of values for each biomarker, with an internal boxplot showing the interquartile range (IQR), the median (white dot), and the data range (excluding outliers). These plots illustrate differences in circulating immune mediator levels between infected and non-infected animals. Values shown correspond to z-score standardized measurements (centered by subtracting the mean and scaled by the standard deviation); negative values indicate measurements below the cohort mean.
(TIFF)

S3 Fig Violin plots of blood parameter levels measured in non-infected chimpanzees and in chimpanzees infected with different *Plasmodium* species combinations. Infection categories include PG/PR/PV (n = 7), PG/PR (n = 5), and PO (n = 1). Non-infected chimpanzees are also shown (n = 14). Hematological parameters were measured in blood samples collected from each animal. ALAT, ASAT, and GGT values are expressed in units per liter (U/L). Cholesterol (Chol), triglycerides (TG), and urea concentrations are reported in mmol/L. Creatinine (creat) is expressed in μmol/L. White blood cells (WBC), platelets (PQT), lymphocytes (Lymph), and monocytes (Mono) are presented in ×10³/mm³. Red blood cell count (RBC) is expressed in ×10⁶/mm³. Hemoglobin (Hb) is reported in g/dL, and hematocrit (Ht) in percentage (%). Neutrophils (Neut) are expressed in ×10⁹/L. Each violin plot illustrates the distribution of values, with internal boxplots representing the median and interquartile range, highlighting differences in blood profiles across infection statuses. Values are z-score standardized (centered to mean = 0 and scaled to standard deviation = 1); negative values indicate measurements below the overall mean and positive values indicate above-average levels.
(TIFF)

S4 Fig Violin plots of eight cytokine and chemokine parameter levels measured in non-infected chimpanzees and in chimpanzees infected with different *Plasmodium* species combinations. Infection categories include PG/PR/PV (n = 7), PG/PR (n = 5), and PO (n = 1). Non-infected chimpanzees are also shown (n = 14). Cytokine and chemokine levels were quantified from circulating plasma samples using a Luminex multiplex assay (ProcartaPlex, Thermo Fisher Scientific) and are reported as median fluorescence intensity (MFI) values. Each violin plot illustrates the distribution of values, with internal boxplots representing the median and interquartile range, highlighting differences in circulating immune profiles across infection groups. Values shown correspond to z-score standardized measurements (centered by subtracting the mean and scaled by the standard deviation); negative values indicate measurements below the cohort mean.
(TIFF)
