# Supplementary material for: Hidden hematological, biochemical and immune costs of asymptomatic malaria infections in semi-wild chimpanzees
Source: PLoS Pathog. 2026 Jun 23;22(6):e1014287. doi: 10.1371/journal.ppat.1014287 (PMC13289926; doi:10.1371/journal.ppat.1014287)
Supplement: S1 File — Including: Table A. Summary of BLAST results for Plasmodium species detection in chimpanzee samples using MinION sequencing. This table presents the results of a BLAST analysis performed on 10,000 randomly selected reads from MinION sequencing data. Each row corresponds to a different chimpanzee sample, and the columns represent the number of reads matching specific Plasmodium species. PCR amplification of the cytochrome B gene was initially used to detect the presence of Plasmodium species, followed by MinION sequencing. Only samples that tested positive for Plasmodium during the initial PCR and qPCR screening (n = 13 of 27 individuals) were subjected to MinION sequencing, which explains why this table includes results for 13 individuals only. The species identified include P. adleri, P. gaboni, P. ovale-like, P. praefalciparum, P. reichenowi, P. vivax-like, P. billcollinsi, P. malariae-like, and P. blacklocki. The “Non-identified” column indicates the number of reads that could not be confidently assigned to any specific Plasmodiumspecies. The final column totals the number of identified reads for each sample. Names followed by an asterisk (*) indicate individuals PCR-positive for P. vivax-like based on the cox1-specific assay. Table B. Impact of demographic and physiological variables on malaria infection status in chimpanzees in Gabon. This table summarizes the statistical tests used to assess whether host demographic variables were associated with Plasmodium infection status (infected vs. non-infected). Continuous variables (age, weight, and body temperature) were compared between groups using Welch’s t-tests, which are robust to unequal variances and sample sizes. Median and interquartile ranges (IQR) are provided to illustrate the distribution of the variables in each group. Categorical variables (sex and blood group) were evaluated using Fisher’s exact tests, which are appropriate for contingency tables with small sample sizes. For each comparison, the test [file ppat.1014287.s001.docx]

**Supplementary Tables**

**Table A.**

**Table B**.

| **Variable** |  | **Test** | **Statistic** | **P-value** | **Infected median (IQR)** | **Non-infected median (IQR)** |
| --- | --- | --- | --- | --- | --- | --- |
| Age (months) |  | Welch t-test | t = -5.01 | 0.000036* | 118 (97-144) | 380 (242.5-489.5) |
| Weight (kg) |  | Welch t-test | t = -1.45 | 0.16 | 36 (35-53.45) | 49.6 (46.7-53.0) |
| Sex |  | Fisher's exact test | - | 0.35 | - | - |
| Blood group |  | Fisher's exact test | - | 0.82 | - | - |
| Temperature (°C) |  | Welch t-test | t = 1.01 | 0.32 | 36.8 (36.5-37.5) | 36.7 (36.35-37.33) |

**Table C.**

| Parameter |  |  |
| --- | --- | --- |
|  | W | P-value |
| White blood cells | 0.936 | 0.095 |
| Red blood cells | 0.960 | 0.367 |
| Platelets | 0.959 | 0.348 |
| Neutrophyls | 0.926 | 0.055 |
| Monocytes | 0.939 | 0.115 |
| Lymphocytes | 0.984 | 0.942 |
| Hemoglobine | 0.967 | 0.521 |
| Hematocryte | 0.980 | 0.852 |
| GGT | 0.707 | <0.001 |
| ASAT | 0.768 | <0.001 |
| ALAT | 0.821 | <0.001 |
| Urea | 0.782 | <0.001 |
| Creatinine | 0.967 | 0.517 |
| Triglycerides | 0.580 | <0.001 |
| Cholesterol | 0.967 | 0.521 |
| IL4 | 0.939 | 0.115 |
| CCL3 | 0.795 | <0.001 |
| CCL5 | 0.953 | 0.248 |
| TNF | 0.871 | 0.003 |
| IL6 | 0.831 | <0.001 |
| IFNG | 0.544 | <0.001 |
| IL1B | 0.718 | <0.001 |
| IL10 | 0.714 | <0.001 |

**Table D.**

| Sample | Infection status | *P. gaboni* reads | *P. reichenowi* reads | *P. ovale-like* reads | *P. vivax-like* PCR | qPCR Ct (Cq) | Parasitemia (parasites/µL) |
| --- | --- | --- | --- | --- | --- | --- | --- |
| Tarzan | Positive | 2465 | 7518 | 2 | Negative | 34.37 | 5.085 |
| Felix | Positive | 1817 | 8141 | 6 | Negative | 23.58 | 727.259 |
| Cerise | Positive | 8114 | 1860 | 4 | Negative | 31.52 | 7.792 |
| Nikita | Positive | 2263 | 7653 | 51 | Negative | 31.54 | 7.749 |
| Charly | Positive | 1759 | 8196 | 5 | Negative | 30.36 | 11.614 |
| Nzigou | Positive | 22 | 94 | 9767 | Negative | 34.3 | 5.107 |
| Tonic | Positive | 8714 | 1034 | 201 | Positive | 27.36 | 59.007 |
| Ogooue | Positive | 6158 | 3798 | 11 | Positive | 22.7 | 1323.857 |
| Ebene | Positive | 5871 | 4106 | 2 | Positive | 26.19 | 125.694 |
| Malimbe | Positive | 7869 | 2107 | 3 | Positive | 24.56 | 374.018 |
| Flore | Positive | 459 | 9524 | 3 | Positive | 19.81 | 9534.063 |
| Wonga | Positive | 5221 | 4652 | 1 | Positive | 21.07 | 4028.724 |
| Ebea | Positive | 3603 | 6356 | 7 | Positive | 21.4 | 3251.087 |
| Dewie | Negative | NA | NA | NA | Negative | 37.4 | 0.062 |
| Raponda | Negative | NA | NA | NA | Negative | 36.2 | 0.112 |
| Moanda | Negative | NA | NA | NA | Negative | 38.1 | 0.043 |
| Adane | Negative | NA | NA | NA | Negative | 36.7 | 0.093 |
| Geraldine | Negative | NA | NA | NA | Negative | 37.9 | 0.05 |
| Mpassa | Negative | NA | NA | NA | Negative | 35.9 | 0.13 |
| Amelie | Negative | NA | NA | NA | Negative | 38.3 | 0.037 |
| Bernard | Negative | NA | NA | NA | Negative | 36.5 | 0.099 |
| Ayrton | Negative | NA | NA | NA | Negative | 37.1 | 0.074 |
| Noirot | Negative | NA | NA | NA | Negative | 38 | 0.05 |
| Charles | Negative | NA | NA | NA | Negative | 36.8 | 0.087 |
| Maguy | Negative | NA | NA | NA | Negative | 37.6 | 0.056 |
| Clara | Negative | NA | NA | NA | Negative | 36 | 0.124 |
| Morphee | Negative | NA | NA | NA | Negative | 38.4 | 0.031 |

**Table E.**
