# Supplementary figures and images for: Hidden hematological, biochemical and immune costs of asymptomatic malaria infections in semi-wild chimpanzees
Source: PLoS Pathog. 2026 Jun 23;22(6):e1014287. doi: 10.1371/journal.ppat.1014287 (PMC13289926; doi:10.1371/journal.ppat.1014287)

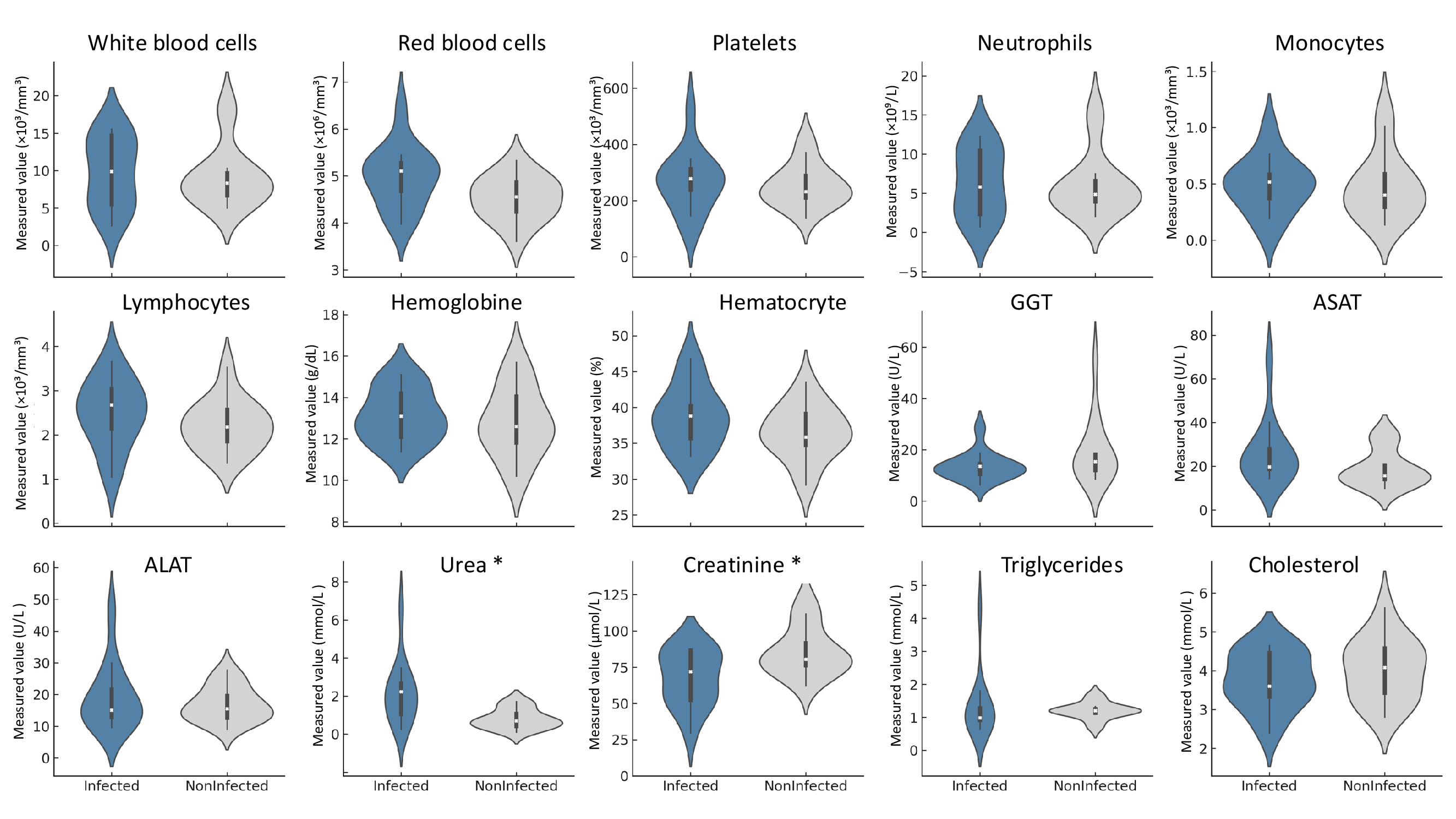

Supplement: S1 Fig — This figure displays the distribution of 15 blood parameters measured in Plasmodium-infected and non-infected chimpanzees. Each violin plot shows the full distribution of values, with an embedded box plot indicating the interquartile range (IQR), the median (central white dot), and the data range (excluding outliers). ALAT, ASAT, and GGT values are expressed in units per liter (U/L). Cholesterol (Chol), triglycerides (TG), and urea concentrations are reported in mmol/L. Creatinine (creat) is expressed in μmol/L. White blood cells (WBC), platelets (PQT), lymphocytes (Lymph), and monocytes (Mono) are presented in ×10³/mm³. Red blood cell count (RBC) is expressed in ×10⁶/mm³. Hemoglobin (Hb) is reported in g/dL, and hematocrit (Ht) in percentage (%). Neutrophils (Neut) are expressed in ×10⁹/L. These plots allow visual comparison of central tendencies and distributional differences in blood markers between infected and non-infected animals, highlighting potential physiological changes associated with Plasmodium infection. Values are z-score standardized (centered to mean = 0 and scaled to standard deviation = 1); negative values indicate measurements below the overall mean and positive values indicate above-average levels. Statistical significance is indicated by asterisks (*P < 0.05). (TIFF) [file ppat.1014287.s002.tiff]

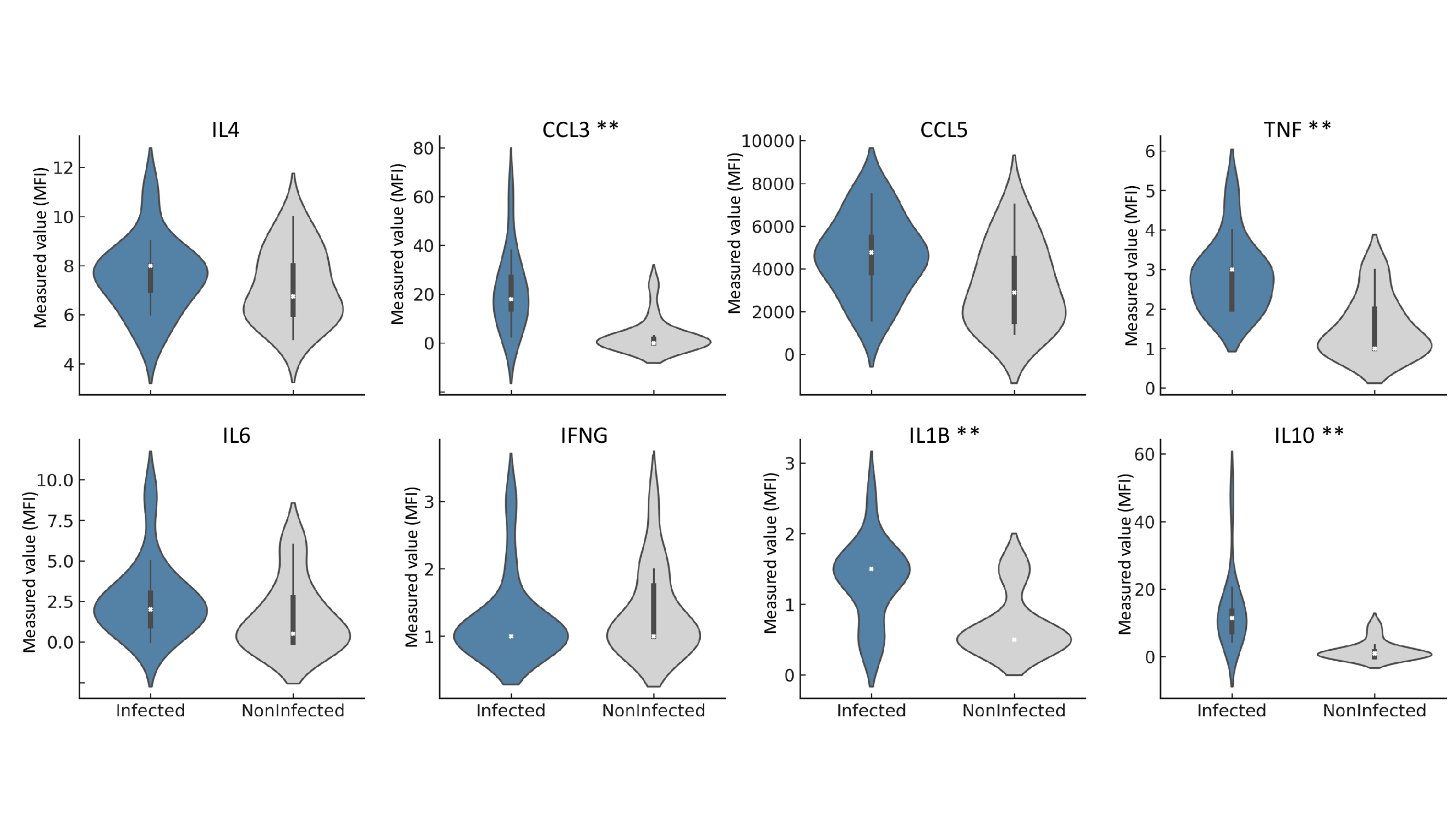

Supplement: S2 Fig — This figure presents the distribution of eight cytokine and chemokine parameters measured in circulating plasma samples from chimpanzees infected with Plasmodium and those that were not infected. Cytokine and chemokine levels were quantified using a Luminex multiplex assay (ProcartaPlex Non-Human Primate Cytokine & Chemokine Panel 30-plex, Thermo Fisher Scientific) and are reported as background-adjusted median fluorescence intensity (MFI) values. Each violin plot displays the full distribution of values for each biomarker, with an internal boxplot showing the interquartile range (IQR), the median (white dot), and the data range (excluding outliers). These plots illustrate differences in circulating immune mediator levels between infected and non-infected animals. Values shown correspond to z-score standardized measurements (centered by subtracting the mean and scaled by the standard deviation); negative values indicate measurements below the cohort mean. (TIFF) [file ppat.1014287.s003.tiff]

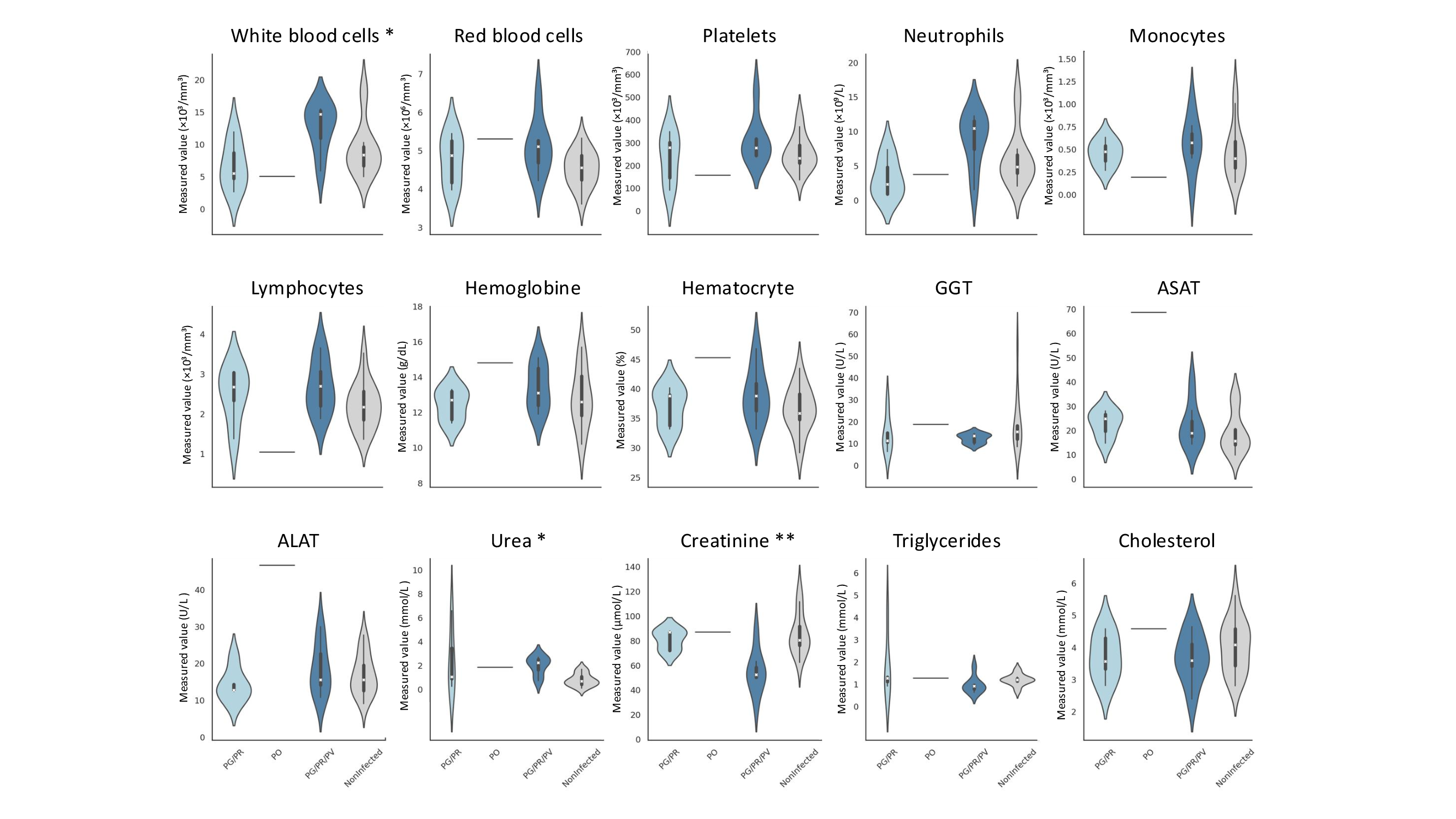

Supplement: S3 Fig — Infection categories include PG/PR/PV (n = 7), PG/PR (n = 5), and PO (n = 1). Non-infected chimpanzees are also shown (n = 14). Hematological parameters were measured in blood samples collected from each animal. ALAT, ASAT, and GGT values are expressed in units per liter (U/L). Cholesterol (Chol), triglycerides (TG), and urea concentrations are reported in mmol/L. Creatinine (creat) is expressed in μmol/L. White blood cells (WBC), platelets (PQT), lymphocytes (Lymph), and monocytes (Mono) are presented in ×10³/mm³. Red blood cell count (RBC) is expressed in ×10⁶/mm³. Hemoglobin (Hb) is reported in g/dL, and hematocrit (Ht) in percentage (%). Neutrophils (Neut) are expressed in ×10⁹/L. Each violin plot illustrates the distribution of values, with internal boxplots representing the median and interquartile range, highlighting differences in blood profiles across infection statuses. Values are z-score standardized (centered to mean = 0 and scaled to standard deviation = 1); negative values indicate measurements below the overall mean and positive values indicate above-average levels. (TIFF) [file ppat.1014287.s004.tiff]

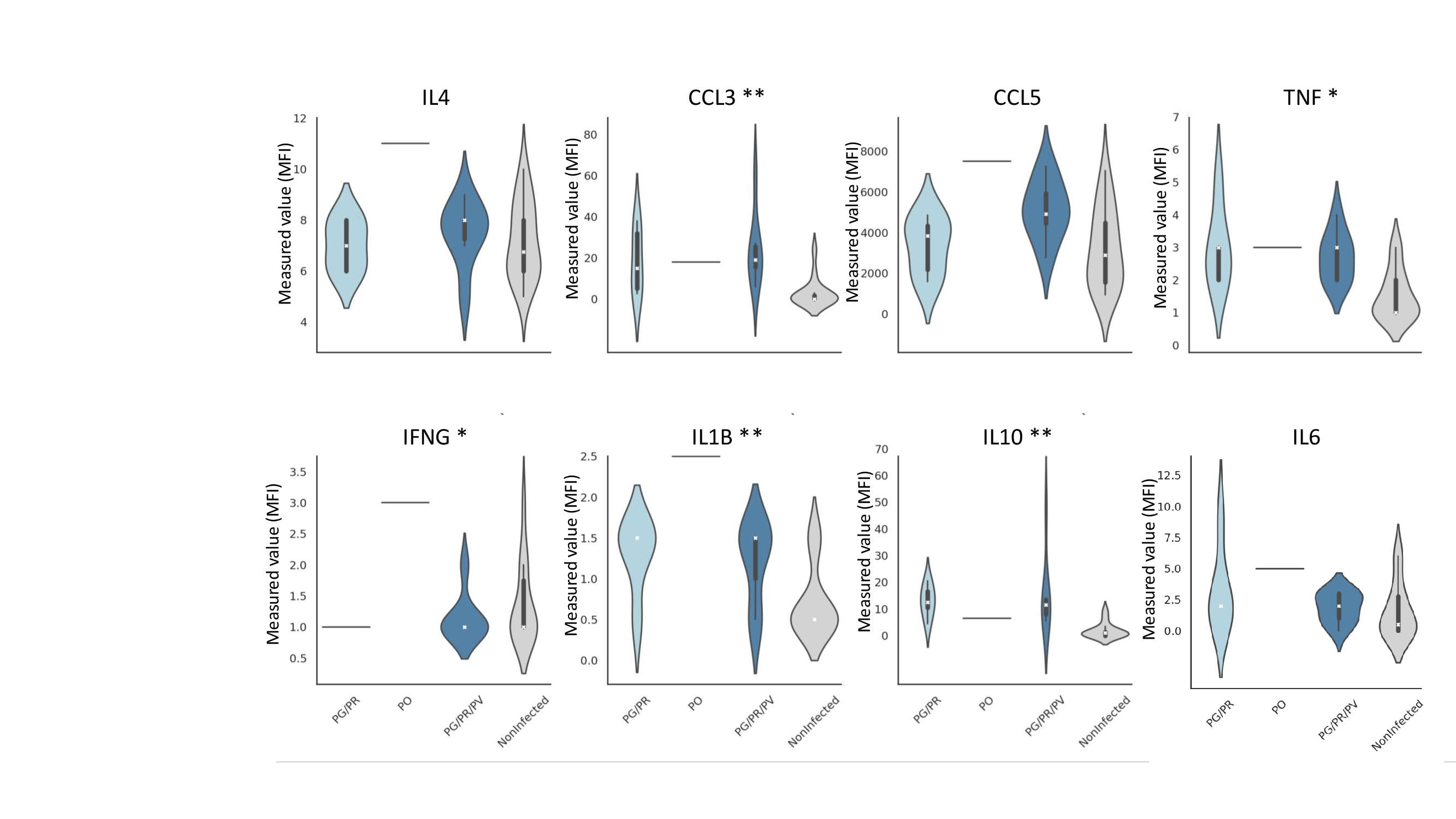

Supplement: S4 Fig — Infection categories include PG/PR/PV (n = 7), PG/PR (n = 5), and PO (n = 1). Non-infected chimpanzees are also shown (n = 14). Cytokine and chemokine levels were quantified from circulating plasma samples using a Luminex multiplex assay (ProcartaPlex, Thermo Fisher Scientific) and are reported as median fluorescence intensity (MFI) values. Each violin plot illustrates the distribution of values, with internal boxplots representing the median and interquartile range, highlighting differences in circulating immune profiles across infection groups. Values shown correspond to z-score standardized measurements (centered by subtracting the mean and scaled by the standard deviation); negative values indicate measurements below the cohort mean. (TIFF) [file ppat.1014287.s005.tiff]
