# Supplementary material for: Hidden hematological, biochemical and immune costs of asymptomatic malaria infections in semi-wild chimpanzees
Source: PLoS Pathog. 2026 Jun 23;22(6):e1014287. doi: 10.1371/journal.ppat.1014287 (PMC13289926; doi:10.1371/journal.ppat.1014287)
Supplement: Attachment — Submitted filename: Answers_Editor_Reviewers_PlosPat_2026.docx [file ppat.1014287.s007.docx]

**REHABS International Research Laboratory**

**Nelson Mandela University**

**George Campus, Madiba Drive,**

**6529 George, South Africa**

**Journal Requirements:**

1) We ask that a manuscript source file is provided at Revision. Please upload your manuscript file as a .doc, .docx, .rtf or .tex. If you are providing a .tex file, please upload it under the item type u2018LaTeX Source Fileu2019 and leave your .pdf version as the item type u2018Manuscriptu2019.

**VR. The revised manuscript is provided as a .docx source file, in accordance with the journal submission requirements.**

2) Please provide an Author Summary. This should appear in your manuscript between the Abstract (if applicable) and the Introduction, and should be 150-200 words long. The aim should be to make your findings accessible to a wide audience that includes both scientists and non-scientists. Sample summaries can be found on our website under Submission Guidelines:
   <https://journals.plos.org/plospathogens/s/submission-guidelines#loc-parts-of-a-submission>

**VR. We thank the editor for this reminder. An Author summary (180 words) has now been added to the revised manuscript between the Abstract and the Introduction, in accordance with the journal’s submission guidelines.**

3) We noticed that you used the phrase 'unpublished data' in the manuscript. We do not allow these references, as the PLOS data access policy requires that all data be either published with the manuscript or made available in a publicly accessible database. Please amend the supplementary material to include the referenced data or remove the references.

**VR. We thank the editor for highlighting this issue. In accordance with the PLOS data access policy, all references to “unpublished data” have been removed from the manuscript. Specifically, the sentence in the Discussion that previously referred to unpublished longitudinal observations has been rewritten to avoid referencing data not presented in the manuscript. In addition, in the Methods section, the wording “custom, unpublished qPCR assay” has been replaced by a full description of the assay and reaction conditions to ensure methodological transparency. These modifications ensure that the manuscript fully complies with the PLOS data availability requirements.**

4) We do not publish any copyright or trademark symbols that usually accompany proprietary names, eg ©,  ®, or TM  (e.g. next to drug or reagent names). Therefore please remove all instances of trademark/copyright symbols throughout the text, including:
   - © on page: 17
   - ® on pages: 14, and 16.

**VR. We thank the editor for pointing this out. In accordance with the journal’s formatting requirements, all trademark and copyright symbols have been removed from the manuscript. Specifically, the symbols associated with proprietary product names have been deleted while retaining the product names themselves for methodological clarity. These corrections have been implemented in the revised manuscript ( for Imalgene, SERAFOL ABO+D kit, and MagPix system).**

5) Please upload all main figures as separate Figure files in .tif or .eps format. For more information about how to convert and format your figure files please see our guidelines:
   <https://journals.plos.org/plospathogens/s/figures>

**VR. We thank the editor for this clarification. In accordance with the journal’s guidelines, all main figures have now been uploaded as separate figure files in .tif format during the revision submission process.**

6) We have noticed that you have uploaded Supporting Information files, but you have not included a list of legends. Please add a full list of legends for your Supporting Information files after the references list.

**VR. We thank the editor for pointing this out. A complete list of Supporting Information legends has now been added to the manuscript after the References list, in accordance with the journal guidelines. The legends correspond to the Supporting Tables and Figures provided as supplementary files.**

7) Some material included in your submission may be copyrighted. According to PLOSu2019s copyright policy, authors who use figures or other material (e.g., graphics, clipart, maps) from another author or copyright holder must demonstrate or obtain permission to publish this material under the Creative Commons Attribution 4.0 International (CC BY 4.0) License used by PLOS journals. Please closely review the details of PLOSu2019s copyright requirements here: PLOS Licenses and Copyright. If you need to request permissions from a copyright holder, you may use PLOS's Copyright Content Permission form.
   Please respond directly to this email and provide any known details concerning your material's license terms and permissions required for reuse, even if you have not yet obtained copyright permissions or are unsure of your material's copyright compatibility. Once you have responded and addressed all other outstanding technical requirements, you may resubmit your manuscript within Editorial Manager. 
   Potential Copyright Issues:
   i) Figure 5. Please confirm whether you drew the images / clip-art within the figure panels by hand. If you did not draw the images, please provide (a) a link to the source of the images or icons and their license / terms of use; or (b) written permission from the copyright holder to publish the images or icons under our CC BY 4.0 license. Alternatively, you may replace the images with open source alternatives. See these open source resources you may use to replace images / clip-art:
   - <https://commons.wikimedia.org>
   - <https://openclipart.org/>.

ii) Figure 1A. Please (a) provide a direct link to the base layer of the map (i.e., the country or region border shape) and ensure this is also included in the figure legend; and (b) provide a link to the terms of use / license information for the base layer image or shapefile. We cannot publish proprietary or copyrighted maps (e.g. Google Maps, Mapquest) and the terms of use for your map base layer must be compatible with our CC BY 4.0 license.
Note: if you created the map in a software program like R or ArcGIS, please locate and indicate the source of the basemap shapefile onto which data has been plotted.
If your map was obtained from a copyrighted source please amend the figure so that the base map used is from an openly available source. Alternatively, please provide explicit written permission from the copyright holder granting you the right to publish the material under our CC BY 4.0 license.
If you are unsure whether you can use a map or not, please do reach out and we will be able to help you. The following websites are good examples of where you can source open access or public domain maps:
* U.S. Geological Survey (USGS) - All maps are in the public domain. (<http://www.usgs.gov>)
* PlaniGlobe - All maps are published under a Creative Commons license so please cite u201cPlaniGlobe, <http://www.planiglobe.com>, CC BY 2.0u201d in the image credit after the caption. (<http://www.planiglobe.com/?lang=enl>)
* Natural Earth - All maps are public domain. (<http://www.naturalearthdata.com/about/terms-of-use/>).

**VR. We thank the editor for raising this point and for highlighting the PLOS copyright requirements. To ensure full compliance with the journal’s licensing policy, we have clarified the sources and licensing information directly in the figure legends. For Figure 1A, we have added a sentence specifying the source of the base maps and their modification by the authors. The legend now includes the following statement: “Base maps of Africa and Gabon were obtained from Wikimedia Commons (https://commons.wikimedia.org/wiki/File:BlankMap-Africa.svg;** [**https://commons.wikimedia.org/wiki/File:Gabon_sat.png**](https://commons.wikimedia.org/wiki/File:Gabon_sat.png)**) and modified by the authors.” For Figure 5, we have clarified the origin of the graphical elements used in the schematic. The legend now includes the following statement:**

**“Human and chimpanzee icons were drawn by the authors.” These additions ensure that the sources and licensing information for all graphical elements are clearly indicated and compatible with publication under the Creative Commons Attribution 4.0 (CC BY 4.0) license used by PLOS journals.**

8) In the online submission form, you indicated that  The raw data supporting the conclusions of this article is available upon request.. All PLOS journals now require all data underlying the findings described in their manuscript to be freely available to other researchers, either
1. In a public repository
2. Within the manuscript itself
3. Uploaded as supplementary information.
This policy applies to all data except where public deposition would breach compliance with the protocol approved by your research ethics board. If your data cannot be made publicly available for ethical or legal reasons (e.g., public availability would compromise patient privacy), please explain your reasons by return email and your exemption request will be escalated to the editor for approval. Your exemption request will be handled independently and will not hold up the peer review process, but will need to be resolved should your manuscript be accepted for publication. One of the Editorial team will then be in touch if there are any issues.

**VR. We thank the editor for pointing this out. The Data Availability statement has been revised to comply with the PLOS data policy. The previous wording indicating that the raw data were “available upon request” has been removed. All data underlying the findings of this study are now available within the manuscript and its Supporting Information files (in Supplementary Table S5). No additional restrictions apply to the availability of the data.**

9) Kindly revise your competing statement in the online submission form to align with the journal's style guidelines: 'The authors declare that there are no competing interests.'

**VR. We thank the editor for pointing this out. The competing interests statement in the online submission form has been revised to follow the journal’s required wording. It now reads: “The authors declare that there are no competing interests.”**

--------------------------------------------------------------------------------------------------------------

**Reviewers' Comments:**

**Part I**

**VR. We would like to first thank both reviewers for their careful evaluation of our manuscript and for their constructive and encouraging comments. We are pleased that the reviewers recognized the originality and importance of this work in improving our understanding of *Plasmodium* infections in great apes. Following their suggestions, we have substantially revised the manuscript to improve clarity, methodological transparency, and statistical rigor.**

**Reviewer #1:** The study by Nowakowski highlights very important and interesting points, as great apes can be infected by malaria parasites, including some species similar to those that infect humans, if I understood correctly. A better understanding of malaria infection in great apes, as demonstrated in the work presented here, is essential to achieving the ultimate goal of eradicating malaria. However, the presentation of the study requires greater rigor, specifically regarding the Luminex data and the statistical tests used. Currently, the methods section lacks sufficient information to allow for reproducibility of the study. I advise the authors to assess the distribution of their data to determine whether the use of parametric tests is appropriate. If this analysis has already been performed, the information should be included in the methods. Regarding the Luminex data, it is unclear which samples were used in each of the two different Luminex kits, and which reported data are from plasma samples versus PBMC-stimulated supernatants. Clarifications on these points will greatly strengthen the manuscript.

**VR** **We thank the reviewer for these constructive comments. In the revised manuscript, we have expanded the Methods section to improve clarity and ensure the reproducibility of the analyses. Specifically, we now provide additional details regarding the statistical framework used, including how data distributions were assessed prior to applying parametric tests and the complementary non-parametric analyses performed to confirm the robustness of the results. We have also clarified the Luminex methodology, including the kits used, the types of samples analyzed (plasma versus PBMC-stimulated supernatants), and the approach used to process cytokine measurements. Figure legends and the Methods section were revised accordingly to clearly distinguish circulating cytokine measurements from PBMC-stimulated responses. Detailed explanations addressing these methodological clarifications are provided in the responses to the specific comments below.**

**Reviewer #2:** The authors present an excellent piece of work representing a unique opportunity to rigorously assess Plasmodium infection in chimpanzees. The conclusions are generally well aligned with the data, and the work is very nearly complete. The authors should be commended on an ambitious and fascinating project. There are a couple deficits which should be corrected to enable full realization of the work including: 1) clarifying true negativity in the uninfected group and; 2) drawing clearer comparisons to human infections in terms of age distribution and acquisition of asymptomatic infection.

**VR. We thank the reviewer for their positive evaluation of our study and for the constructive suggestions that helped strengthen the manuscript. In the revised version, we addressed the reviewer’s concerns in two main ways. First, we clarified the definition of true negativity in the non-infected group by providing additional details on the molecular screening procedures used to classify individuals as uninfected. In particular, we now report the qPCR results for all chimpanzee samples, including those that were negative by conventional PCR and therefore not selected for nanopore sequencing. These results are presented in a newly added Supplementary Table S4, which reports cycle threshold (Ct) values and corresponding parasitemia estimates for all individuals, providing a clearer overview of infection detection across the cohort. Second, we expanded the interpretation of infection patterns in relation to human malaria, particularly with respect to age-related differences in infection dynamics and the interpretation of infections associated with very low parasite densities. The revised manuscript now places these results in a broader comparative context, including discussion of how parasite density, physiological markers, and immune responses observed in chimpanzees relate to patterns described in human malaria infections. Finally, we clarified the interpretation of infection status and physiological responses by adding comparisons with published reference ranges for chimpanzee hematological and biochemical parameters, and by discussing limitations related to the age structure of the cohort, particularly the limited representation of young individuals. Detailed explanations of these revisions are provided in the responses to the specific comments below.**

--------------------------------------------------------------------------------------------------------------

**Part II – Major Issues: Key Experiments Required for Acceptance**

**Reviewer #1:** 

- The Luminex assays are not clear. You mention 2 kits (one from Invitrogen and one from BioTechne), are they different? If yes, do you mean that you use one kit for the plasma sample and the other one for the supernatant of the in vitro stimulation assay? Can you please give the proper reference to these kits? As of now, using only the name you cited is not enough to find a kit. Did you compare data generated from these 2 kits? How did you correct for possible batch effect? In the manuscript, sometimes you mentioned MFI and sometimes quantitative data. Can you please expand on how the data from these 2 kits were analyze and if any quantitative analysis were performed, how you did. Also, how much volume did you use? at which dilutions? Singlet or duplicate? Luminex kits usually use different dilution of the plasma versus the supernatants. As of now, we do not know where the data shown in the figures are coming from (plasma vs supernatant). It is crucial to better indicate which analysis you did using the plasma data versus the supernatants from the PMA/Iono stimulations of the PBMCs as is can mislead the interpretation.

**VR. We thank the reviewer for this important comment and apologize for the lack of clarity in the original Methods description. The Luminex assay section has now been substantially revised to provide detailed methodological information and improve reproducibility (Methods, lines 501–629). First, we clarify that only a single multiplex cytokine assay kit was used throughout the study, namely the *ProcartaPlex™ Non-Human Primate Cytokine & Chemokine Panel 30-plex* (Thermo Fisher Scientific / Invitrogen; catalogue EPX300-40044-901). We apologize for the confusion caused by the previous mention of BioTechne, which referred only to the Luminex detection platform, not to a second cytokine assay kit. This ambiguity has now been removed from the Methods section. For each chimpanzee, three sample types were analysed on the same assay plate: one circulating plasma sample representing baseline cytokine levels, one PMA-stimulated PBMC supernatant, and one ionomycin-stimulated PBMC supernatant. All samples from the same individual were therefore processed on the same plate, minimizing potential inter-plate variability and eliminating the need for batch normalization procedures. Assays were performed according to the manufacturer’s protocol using a total reaction volume of 50 μL. Plasma samples were analysed using 25 μL of undiluted plasma, while 50 μL of undiluted PBMC supernatant was used for stimulated samples, following the manufacturer’s recommended protocol. All samples were run in singlicate. Cytokine and chemokine responses were analysed using background-adjusted median fluorescence intensity (MFI) values rather than interpolated concentrations derived from standard curves. This approach was used because several analytes fell below the lower limit of quantification of the supplied standards, which would otherwise result in values reported only as below detection. Using MFI values preserves signal resolution across low-response samples and allows more robust comparisons among individuals. This analytical strategy is commonly applied in malaria immunology and sero-epidemiological studies when reliable concentration estimates cannot be obtained from standard curves, and supporting references have been added to the Methods section (Wu et al., 2020; Macalinao et al., 2023). Finally, to avoid ambiguity regarding sample origin, figure legends and table captions have been revised to clearly distinguish circulating cytokine measurements obtained from plasma from cytokine responses measured in stimulated PBMC supernatants (Tables 1–4; Figure 3; Figure 4; Supplementary Figures S2 and S4).**

**References :**

**Wu L, Hall T, Ssewanyana I, Oulton T, Patterson C, Vasileva H, Singh S, Affara M, Mwesigwa J, Correa S, Bah M, D&#39;Alessandro U, Sepúlveda N, Drakeley C, Tetteh KKA. Optimisation and standardisation of a multiplex immunoassay of diverse Plasmodium falciparum antigens to assess changes in malaria transmission using sero- epidemiology. Wellcome Open Res. 2020 Apr 23;4:26. doi: 10.12688/wellcomeopenres.14950.2. PMID: 32518839; PMCID: PMC7255915.**

**Macalinao MLM, Fornace KM, Reyes RA, Hall T, Bareng APN, Adams JH, Huon C, Chitnis CE, Luchavez JS, Tetteh KKA, Yui K, Hafalla JCR, Espino FEJ, Drakeley CJ. Analytical approaches for antimalarial antibody responses to confirm historical and recent malaria transmission: an example from the Philippines. Lancet Reg Health West Pac. 2023 May 20;37:100792. doi: 10.1016/j.lanwpc.2023.100792. PMID: 37693871; PMCID: PMC10485684.**

- Welch’s test is a parametric test. Did you assess the distribution of the data prior selecting parametric test? If not, you should assess the distribution of your data prior selecting a test. Data that are not following a normal distribution should use a non-parametric test. I will suggest to the authors to review all their data distribution and tests. Line 517-519: What do you mean by “standardized using z-scores”? what did you standardized exactly? Please add details.

**VR. We thank the reviewer for this important comment. We agree that assessing the distribution of the data prior to selecting appropriate statistical tests is essential, and we have clarified this procedure that we applied in the manuscript. Prior to performing group comparisons, the distribution of each continuous physiological variable (hematological parameters, biochemical markers, and cytokine measurements) was assessed using Shapiro–Wilk normality tests and visual inspection of Q–Q plots, as now described in the *Statistical Analyses* section of the Methods. The results of these normality tests are provided in Supplementary Table S3. As shown in this table, several variables, particularly biochemical markers and cytokine measurements, show moderate deviations from strict normality, which is common for physiological datasets. Because sample sizes were relatively small and several variables also showed unequal variances between groups, we used Welch’s t-tests for group comparisons. Welch’s test is robust to heteroscedasticity and moderate deviations from normality and is therefore appropriate under these conditions. The description and justification of this approach have now been added to the Methods section (lines 630-693). We have also clarified the description of z-score standardization (lines 636-703). In the revised manuscript, this refers to the transformation applied to continuous physiological variables before multivariate analyses. Specifically, each variable was centered by subtracting its mean and scaled by dividing by its standard deviation, resulting in variables with mean = 0 and standard deviation = 1. This transformation ensures that parameters measured on different numerical scales contribute equally to multivariate analyses and prevents variables with larger numeric ranges from disproportionately influencing the results. This standardization was applied prior to Principal Component Analysis (PCA) and penalized logistic regression (LASSO), and the Methods section has been revised accordingly.**

**Reviewer #2:**

1. Can the authors include qPCR data for the nanopore-negative samples as well? This will not only help ensure accuracy of the nanopore, but strengthen arguments about asymptomatic infection.

**VR. We thank the reviewer for this very helpful suggestion. In response, we have now included the qPCR results for all chimpanzee samples analysed in this study, including those that were negative for *Plasmodium* detection by conventional PCR and therefore not subjected to nanopore sequencing. These results are presented in the newly added Supplementary Table S4, which reports, for each individual, the qPCR cycle threshold (Ct) values and the corresponding estimated parasitemia (parasites/µL). For samples identified as *Plasmodium*-positive, Table S4 also reports the MinION sequencing results (number of reads assigned to *P. gaboni*, *P. reichenowi*, and *P. ovale*-like parasites) as well as the result of the species-specific *P. vivax*-like PCR assay. To clarify the sequencing workflow, the revised manuscript now explicitly states that only samples positive by both nested PCR and qPCR were selected for MinION sequencing, while samples negative by these molecular screening assays were not sequenced (lines 548-551). This approach was implemented to optimize sequencing resources by focusing high-throughput sequencing on samples with confirmed parasite DNA. Indeed, for individuals classified as *Plasmodium*-negative, qPCR Ct values were generally high (Ct ≈ 35–38), corresponding to extremely low estimated parasite densities close to the detection limit of the assay. We agree that reporting these values provides a clearer overview of infection detection across the cohort and further supports the classification of individuals as infected or non-infected (Supplementary Table S4). The Methods and Results sections (lines 520-551; lines 200-208 respectively) have been revised to describe the criteria used for selecting samples for nanopore sequencing and to reference the qPCR results reported in Supplementary Table S4.**

2. For the blood parameters in Tables 1 and 2 as well as Figure 3, could the authors provide some context of normal ranges. I understand these may be speculative for chimpanzees, but rough reference values should be available. This will help interpret what, if any, of these deviations are clinically significant.

**VR. We thank the reviewer for this helpful suggestion. To provide biological context for the hematological and biochemical values reported in Tables 1 and 2 and Figure 3, we have added comparisons with previously published physiological reference ranges for chimpanzees in the revised manuscript. Specifically, we now refer to the dataset reported by Howell et al. (2003), which established hematologic and serum chemistry reference values for healthy captive chimpanzees (*Pan troglodytes*). The manuscript now explicitly states that the haematological and biochemical parameters measured in this study fall within the physiological ranges previously reported for the species (lines 582-586). In the Discussion (lines 359-366), we also clarify the biological interpretation of the observed differences between infected and non-infected individuals. Although several parameters differed statistically between groups (notably urea, creatinine, and several cytokine markers), their absolute values remained within the physiological ranges reported for chimpanzees, suggesting that the infections detected in this population are associated with relatively low parasite densities and limited clinical impact. This interpretation is consistent with the largely subclinical nature of many *Laverania* infections reported in African great apes.**

**Reference:
Howell S., Hoffman K., Bartel L., Schwandt M., Morris J., Fritz J. (2003). *Normal hematologic and serum clinical chemistry values for captive chimpanzees (Pan troglodytes).* Comparative Medicine 53:413–423.**

3. The effect of age is a very interesting and important finding, yet needs to be more concrete. Since there is only a single animal under ~7 years of age, this should be a major caveat discussed considering that the heaviest burden of infection and disease in humans occurs in the first few years of life. Secondly, to claim that the older animals are uninfected, qPCR would be needed.

**VR. We thank the reviewer for this important comment. We agree that the age structure of our dataset represents a potential limitation for interpreting age-related infection patterns. In our cohort, only two individuals were younger than seven years of age, and only one of these animals was *Plasmodium*-infected. Because of this limited representation of juvenile individuals, we have revised the Results (lines 227-233) and Discussion (lines 323-333; lines 344-352) sections to explicitly acknowledge this limitation and to interpret the observed association between age and infection status cautiously. The manuscript now states that parasitemia tended to be higher in younger animals based on regression analyses, but that this pattern should be interpreted carefully, given the small number of juvenile individuals in the dataset. To address the reviewer’s second point regarding infection status in older individuals, we have now included qPCR results for all chimpanzee samples, including those that were negative by conventional PCR and therefore not selected for nanopore sequencing. These results are reported in the newly added Supplementary Table S4, which provides, for each individual, the qPCR cycle threshold (Ct) values and the corresponding parasitemia estimates, together with MinION sequencing results where available and the species-specific *P. vivax*-like PCR results. For individuals classified as non-infected, qPCR Ct values were generally high (Ct ≈ 35–38), corresponding to extremely low estimated parasite densities close to the detection limit of the assay. These results support the classification of these individuals as non-infected based on molecular screening, while acknowledging that very low parasite DNA levels may occasionally occur near the detection threshold.**

4. The unpublished longitudinal data mentioned in the Discussion should either be presented to add context or removed.

**VR. We thank the reviewer for highlighting this issue. In accordance with the editor’s recommendation and the PLOS data availability policy, we have removed all references to unpublished data from the manuscript. The sentence in the Discussion that previously referred to unpublished longitudinal observations has been revised to eliminate any mention of data not presented in the study. These modifications ensure that the manuscript no longer refers to unpublished datasets and that all interpretations are based exclusively on the data presented in the manuscript and its supplementary materials, fully complying with the PLOS data availability requirements.**

5. Non-human primates, at least those in captive colonies, mask symptoms and pain extremely well especially in the presence of humans. Considering this and that animals were sedated during collection, discussion of “asymptomatic” infection needs to be adjusted and supported by more data. In humans, symptomatic malaria includes positivity (often by smear) and fever, chills, etc. Obviously, symptoms outside of fever are impossible to assess. Therefore, it would be more useful if temperature is included (if measured) and the discussion centered around comparisons of objective values obtained from blood paramaters to not just normal ranges but also those seen for malaria infections in human across the ages (from symptomatic children to asymptomatic adults). This is done to some extent for the inflammatory cytokines, but more context via comparisons to healthy and unhealthy values would greatly improve interpretability.

**VR. We thank the reviewer for this important comment and agree that clinical symptoms are difficult to assess in non-human primates, particularly during routine veterinary procedures where animals are sedated and behavioural signs cannot be reliably evaluated. We have therefore revised the manuscript to avoid over-interpreting the clinical status of infected individuals. Specifically, the wording referring to “asymptomatic infections” has been adjusted throughout the manuscript to clarify that our interpretation is based primarily on objective physiological measurements, including parasite densities, hematological parameters, biochemical markers, cytokine responses, and body temperature (lines 214-216; lines 314-317). Body temperature was measured during sampling and included in the statistical analyses. No significant differences in temperature were observed between infected and non-infected chimpanzees (Supplementary Table 2), providing no evidence of fever associated with infection in this cohort. In the revised Discussion, we further clarify that although several individuals were infected with *Plasmodium* species, the measured haematological and biochemical values generally remained within the physiological ranges reported for healthy chimpanzees (Howell et al., 2003; lines 305-307; lines 359-361). These observations suggest a limited detectable physiological disturbance associated with infection in this dataset. We also explicitly acknowledge that clinical manifestations such as chills, malaise, or pain cannot be reliably evaluated under these sampling conditions (lines 375-380). Consequently, our interpretation focuses on measurable biological indicators rather than clinical symptomatology. Finally, we expanded the contextual interpretation of both physiological and immunological markers by comparing our observations with patterns described in human malaria. In humans, symptomatic malaria is typically associated with high parasite densities, fever, and pronounced inflammatory responses, whereas individuals with repeated exposure may harbour infections with lower parasite densities and more regulated cytokine responses. We hope these comparisons provide additional context for interpreting the relatively moderate physiological alterations observed in the chimpanzees analysed in this study.**

 --------------------------------------------------------------------------------------------------------------

**Part III – Minor Issues:**

**Reviewer #1:**

Introduction:
- Are these plasmodium species carried by the same vector than the one infecting human?

**VR. We thank the reviewer for this question. Both human and great ape *Plasmodium* parasites are transmitted by mosquitoes of the genus *Anopheles*. However, increasing evidence indicates that malaria parasites infecting African great apes are primarily transmitted by *Anopheles* species adapted to sylvatic forest environments, rather than by the major human malaria vectors such as *Anopheles gambiae* or *Anopheles funestus*. To clarify this point, we have added a sentence in the Introduction indicating that although ape and human malaria parasites share the same mosquito genus (*Anopheles*), their transmission cycles likely involve distinct mosquito species associated with wildlife transmission systems in forest ecosystems (lines 90-95).**

- Do you consider the great apes as potential reservoir for malaria?

**VR. We thank the reviewer for raising this point. African great apes are natural hosts of several *Plasmodium* species closely related to those infecting humans, particularly within the subgenus *Laverania*. Phylogenetic studies have demonstrated that *Plasmodium falciparum*, the main human malaria parasite, originated from a parasite lineage infecting western gorillas, highlighting the evolutionary connections between ape and human malaria parasites. However, current evidence indicates that most ape *Plasmodium* parasites circulate within sylvatic transmission cycles involving ape hosts and forest-associated *Anopheles* vectors, and these parasite lineages appear largely host-specific. While host-switch events have occurred, there is currently limited evidence that African great apes act as active reservoirs sustaining ongoing malaria transmission in human populations. To clarify this distinction, we have revised the Introduction (lines 90-106) to indicate that African great apes represent natural hosts for a diverse assemblage of *Plasmodium* parasites and may constitute sylvatic reservoirs for these parasite lineages, while emphasizing that their contribution to contemporary human malaria epidemiology appears limited based on current evidence.**

- Did you find similar species in the Plasmodium infecting Great Apes and Human in Gabon?

**VR. We thank the reviewer for this important question. Previous studies conducted in Central Africa, including Gabon, have shown that African great apes harbor several *Plasmodium* species closely related to those infecting humans. In particular, chimpanzees and gorillas host multiple members of the subgenus *Laverania*, such as *Plasmodium gaboni* and *P. reichenowi*, which are phylogenetically closely related to human *P. falciparum* (Boundenga et al., 2015). Evolutionary analyses further demonstrated that the diversity of ape *Laverania* parasites represents the ancestral pool from which human *P. falciparum* emerged following a historical cross-species transmission event from gorillas (Rougeron et al., 2022). Although these parasites are generally considered host-specific in natural ecosystems, studies conducted in Gabon have shown that host specificity can occasionally be broken under confined conditions. For example, Ngoubangoye et al. (2016) documented several cross-species transmission events of *Plasmodium* parasites between gorillas, chimpanzees, and other primates living in sanctuaries, as well as rare transfers from humans to captive primates. These findings suggest that ecological conditions bringing different host species into close contact may facilitate parasite exchanges. However, studies investigating potential cross-species transmission in natural settings suggest that ape and human malaria parasite populations remain largely separated. For example, Délicat-Loembet et al. (2015) screened more than 400 human blood samples from individuals living in close proximity to great ape populations in Gabon and detected only classical human malaria parasites (*P. falciparum*, *P. malariae*, and *P. ovale*), with no evidence of ape-derived *Laverania* infections. Our results are consistent with these previous findings. In this study, we detected several ape-associated *Plasmodium*species in chimpanzees (*P. gaboni*, *P. reichenowi*, and *P. vivax*-like), which belong to parasite lineages naturally circulating in African great apes. We did not detect evidence suggesting transmission of these parasites to humans. We added some sentences in the Introduction to highlight this (lines 90-106).**

**References added:**

**Boundenga L. et al., 2015. *Plasmodium species circulating in wild chimpanzees in Gabon.* Parasites & Vectors. https://doi.org/10.1186/s13071-015-0752-4**

**Délicat-Loembet L. et al., 2015. *No evidence for ape Laverania malaria parasites in humans living in Gabon.* PLoS ONE. https://doi.org/10.1371/journal.pone.0126933**

**Rougeron V. et al., 2022. *A population genetic perspective on the origin, spread and adaptation of the human malaria agents Plasmodium falciparum and Plasmodium vivax.* FEMS Microbiology Reviews.** [**https://doi.org/10.1093/femsre/fuab047**](https://doi.org/10.1093/femsre/fuab047)

**Ngoubangoye B. et al. 2016.
The host specificity of ape malaria parasites can be broken in confined environments.
*International Journal for Parasitology.*
https://doi.org/10.1016/j.ijpara.2016.06.004**

Methods:
- For how many individuals the CBC was unavailable?

**VR. We thank the reviewer for this clarification request. Complete blood count (CBC) measurements were available for all chimpanzees included in the study (n = 27), and no individuals were missing CBC data. CBC analyses were performed within 90 minutes of blood collection at the CIRMF Medical Analysis Center, as described in the Methods section of the revised manuscript (lines 584-589).**

- PBMCs stimulation section: how many cells did you stimulate per individuals? And in which final volume of culture media? This information matters as you are using the supernatant to assess the cytokines/chemokines. Adding these details will allow the reproducibility of your data.

**VR. We thank the reviewer for highlighting the importance of providing these methodological details. We have revised the PBMC stimulation section of the Methods to clarify the experimental conditions and improve reproducibility. Specifically, PBMCs isolated from each chimpanzee were cultured in complete RPMI medium in 96-well plates, with 1 × 10⁵ PBMCs per well in a final volume of 200 μL. Cells were stimulated either with phorbol myristate acetate (PMA, 25 ng/mL) or ionomycin (1 μg/mL) for six hours. Following incubation, culture supernatants were collected and stored at −80 °C prior to cytokine and chemokine quantification using a Luminex multiplex assay. To further clarify the experimental design, we now specify that three types of samples were analyzed for each chimpanzee on the same assay plate: one circulating plasma sample representing baseline cytokine levels, one PMA-stimulated PBMC supernatant, and one ionomycin-stimulated PBMC supernatant (lines 610-612).**

- Line 501-502: cytokines/chemokines were assessed from the plasma sample and the supernatants of the stimulated PBMCs. Please correct as of now it sounds that you tested plasma from stimulated PBMCs which is not what it is.

**VR. We thank the reviewer for pointing out this lack of clarity. We agree that the original wording was ambiguous and could incorrectly suggest that plasma was obtained from stimulated PBMCs. The Methods section has therefore been revised to clearly distinguish between circulating plasma samples collected directly from blood and supernatants obtained after ex vivo stimulation of PBMCs. The revised text now specifies that, for each chimpanzee, three distinct sample types were analyzed: (i) one circulating plasma sample representing baseline cytokine levels, and (ii) two PBMC culture supernatants obtained after stimulation with PMA or ionomycin. These samples were processed and analyzed separately for cytokine and chemokine quantification using the Luminex multiplex assay. This clarification has been incorporated into the Methods section to avoid any ambiguity regarding the origin of the samples analyzed.**

- Data availability: you should consider sharing your Luminex and other data through the ImmPort portal.

**VR. We thank the reviewer for this helpful suggestion. In accordance with the PLOS data availability policy, we have ensured that all data underlying the findings of this study are publicly accessible. The complete dataset, including hematological, biochemical, parasitological, and cytokine measurements (including the Luminex data), has been compiled and provided as Supplementary Table S5, which contains the individual-level measurements used in all analyses presented in the manuscript. Because all datasets are now freely available within the manuscript and its Supporting Information files, they can be directly accessed and reused by other researchers without restriction. The Data Availability statement has been revised accordingly to clarify that the complete dataset is provided in Supplementary Table S5.**

Results:
- It is not clear how many chimpanzees were infected versus non-infected and how this determination was made: qPCR results only? Or did you include other parameters such as anemia, fever?

**VR. We thank the reviewer for pointing out this lack of clarity. Infection status in this study was determined exclusively on the basis of molecular detection of *Plasmodium* DNA and qPCR, and not from Minion sequencing data or clinical or physiological parameters (lines 185-189). Specifically, chimpanzee blood samples were screened using PCR targeting the *Plasmodium* cytochrome-b (*cyt-b*) gene, and qPCR was used to quantify parasite density. Only samples positive by both PCR and qPCR were subsequently selected for MinION sequencing to determine *Plasmodium* species composition. Based on this molecular screening strategy, 13 of the 27 chimpanzees (48.15%) were classified as infected, whereas 14 were classified as non-infected. Clinical and physiological parameters, including body temperature, hematological markers, and biochemical measurements, were not used to define infection status, but were analyzed separately to assess potential correlates of *Plasmodium* infection.**

- The tables will benefit to have the age (median/IQR) for each compared set of groups as it seems from the figure 1B that most of the non-infected are older than the infected ones.

**VR. We thank the reviewer for this helpful suggestion. Because age appeared to differ between infected and non-infected chimpanzees (as illustrated in Figure 1B), we have now added the median age and interquartile range (IQR) for each compared group at the top of Tables 1–4. This allows readers to directly visualize the age distribution of the compared groups and facilitates interpretation of the physiological and immunological differences observed between infected and non-infected individuals.**

- Did you correct by age for the analysis as it seems to be a cofounding factor?

**VR. We thank the reviewer for this observation. Because infected chimpanzees were, on average, younger than non-infected individuals, age was considered as a potential confounding factor in our analyses. Age was therefore included as a covariate in the regression-based analyses used to investigate associations between Plasmodium infection and physiological parameters. In particular, age was incorporated into the penalized logistic regression (LASSO) model used to identify predictors of infection status. In addition, the age distribution of the compared groups has now been explicitly reported in the tables (median and interquartile range) to allow readers to evaluate potential demographic differences between infected and non-infected individuals. The Methods section has been clarified accordingly (lines 669-673).**

- It will help to know the number of individuals for each group (PG/PR, PO, …), the only information is 1 individual with Po.

**VR. We thank the reviewer for this helpful suggestion. The number of individuals in each infection category has now been explicitly indicated throughout the manuscript to improve clarity. Sample sizes for each infection group are now reported in the Results section and in the relevant figure and table legends (Figures 1–3, Supplementary Figures S3–S4, and Table 2). The distribution of individuals across infection categories is as follows: PG/PR/PV (n = 7), PG/PR (n = 5), PO (n = 1), and non-infected (n = 14). This clarification ensures that the sample sizes underlying each comparison are clearly visible to the reader.**

- Figure 3, it is not clear if the cytokines/chemokines data are coming from the plasma or from the supernatants of the PBMCs in vitro stimulation assay. Did the authors compare the levels of cytokines/chemokines between the plasma of infected and non-infected individuals, but also between the plasma and supernatants for each group? Because PMA/Ionomycin is a “hammer: triggering high levels of cytokines responses it is not malaria specific. However, comparing the data from the plasma versus the in vitro assay will allow to better be found correlation between the cytokines that are specifically induced during malaria infections.

**VR. We thank the reviewer for this comment and apologize for the lack of clarity. The cytokine and chemokine data shown in Figure 3 correspond exclusively to circulating plasma measurements, quantified using the Luminex multiplex assay, and therefore represent the baseline systemic cytokine profile of each chimpanzee at the time of sampling. The PBMC stimulation assays (PMA and ionomycin) were analyzed separately and are presented in Tables 3 and 4and in the corresponding Results section (“Ex vivo cytokine responses in infected chimpanzees are predominantly anti-inflammatory”). These assays were designed to evaluate the capacity of immune cells to produce cytokines after ex vivo stimulation, rather than circulating cytokine concentrations. Because PMA and ionomycin induce strong non-specific immune activation, plasma cytokine measurements and PBMC stimulation responses were analyzed independently, and no direct comparison between plasma and stimulated cytokine levels was performed. To avoid confusion, we have revised the Figure 3 legend and clarified in the Methods section that Figure 3 reports plasma cytokine measurements, while PBMC stimulation results are presented separately.**

- Missing Y-axis on all the figure 3

**VR. We thank the reviewer for bringing this to our attention. The Y-axis labels have now been carefully checked and clearly added to all panels of Figure 3 in the revised version of the manuscript. The updated figure with complete axis labels has been uploaded to ensure that all measured parameters and units are clearly indicated.**

- Figure 4C, what means “value” in the Y-axis?

**VR. We thank the reviewer for pointing out this lack of clarity. In Figure 4C, the Y-axis represents the observed values of the physiological and cytokine parameters retained as predictors by the LASSO regression model. Because these variables are expressed in different units (e.g., urea in mmol/L and cytokines as median fluorescence intensity, MFI), the axis was initially labeled generically as “value.” To improve clarity, we have revised the figure and updated the Y-axis label to “Measured value.” The figure legend has also been updated to explicitly indicate the units corresponding to each parameter. The corrected Figure 4C has been uploaded in the revised manuscript.**

- The figures will benefit to have the statistical test outcomes indicated.

**VR. We thank the reviewer for this helpful suggestion. The statistical results corresponding to the physiological and cytokine parameters were originally reported in detail in Tables. To improve the readability of the figures and facilitate interpretation without requiring readers to refer to the tables, we have now added statistical significance annotations (asterisks) directly to the relevant panels in the Figure 3 and supplementary Figures 1-4. These asterisks indicate statistically significant differences between groups (P < 0.05). The corresponding statistical tests and exact P-values remain fully reported in Tables 1 and 2. The figure legends have also been updated to clarify the meaning of the significance annotations.**

Discussion:
- Line 246: You mentioned that having more malaria infection in youngling vs adults in chimpanzees’ contrast with human malaria. However, in human, malaria (symptomatic and asymptomatic) is found more frequently in children living in high malaria transmission area compared to the adults. I am not sure I am getting your point. Can you develop your thoughts?

**VR. We thank the reviewer for this important clarification. Our intention was not to suggest that the age pattern observed in chimpanzees contrasts with that described in humans. Indeed, in human populations living in high-transmission areas, malaria infections and parasite densities are typically higher in children, while repeated exposure leads to the gradual acquisition of partial, non-sterilizing immunity in adults that reduces parasite burden and disease severity. In our dataset, infections were also primarily detected in younger chimpanzees, while no older adults tested positive. We have therefore revised the text to clarify that this pattern is broadly consistent with the age-dependent acquisition of parasite control described in humans, rather than opposite to it. However, because our study is cross-sectional and based on a limited sample size, we now emphasize that this interpretation remains tentative and may also reflect sampling effects, differences in exposure, or infections below molecular detection thresholds. The Discussion has been revised accordingly (lines 329-339).**

- Line 289: It is complicated to make a point with “unpublished data” or “data not shown”. Is it possible to add a figure with this data?

**VR. We thank the reviewer for this important comment. In accordance with the reviewer’s suggestion and the PLOS data availability policy, all references to “unpublished data” or “data not shown” have been removed from the manuscript. The sentence in the Discussion that previously referred to unpublished longitudinal observations has been modified to avoid referencing data not presented in the study. The Discussion now relies exclusively on results generated in this study or on previously published work, and no additional figure is therefore required.**

- In your interpretation of the cytokines/chemokines, please indicate where these data come from, the plasma or the stimulated PBMCs, as they are not bringing the same type of information which can mislead the interpretation.

**VR. We thank the reviewer for this important comment. We agree that circulating cytokine concentrations measured in plasma and cytokine production measured after PBMC stimulation reflect different aspects of immune function. Plasma cytokines represent systemic inflammatory or regulatory signals present in circulation, whereas PBMC stimulation assays assess the functional capacity of immune cells to produce cytokines upon activation.
To avoid potential confusion, we have revised the Discussion to clearly distinguish between these two sources of data. Specifically, we now explicitly indicate when cytokine measurements refer to circulating plasma levels (e.g., TNF, IL-10, CCL3, IL-1β) and when they correspond to cytokine production by stimulated PBMCs. These clarifications have been added in the relevant paragraphs of the Discussion.**

Supplementary:
- Supplementary table 1: why there are only the results for 13 individuals? Were they 13 individuals infected with malaria and 14 individuals non-infected (total of 27 individuals)? They are no individuals’ names followed by an asterisk; does it mean that none of them were infected with P. vivax-like? Is it based on this assay that you categorized your “infected” vs “non-infected group”? If so, please add a section about it in the methods.`

**VR. We thank the reviewer for this comment and for the opportunity to clarify this point. Supplementary Table S1 reports the MinION sequencing results, which were generated only for samples that tested positive for *Plasmodium* during the initial molecular screening. In total, 27 chimpanzee blood samples were screened using nested PCR targeting the *Plasmodium*cytochrome-b gene and quantitative PCR (qPCR). Based on these assays, 13 individuals were classified as infected and 14 as non-infected. Only the 13 positive samples were subsequently subjected to nanopore (MinION) sequencing to determine the *Plasmodium* species composition, which explains why Supplementary Table S1 contains results for 13 individuals only. To clarify this workflow, we have expanded the description of the molecular diagnostic pipeline in the Methods section and added a new Supplementary Table S4 reporting the qPCR Ct values and corresponding parasitemia estimates for all 27 individuals.**

- Supplementary table 2: Please indicate if you assessed the distribution of your data to justify the use of the t-test (which one did you used?). The table will benefit of having the median (IQR) values to show the distribution of the data that the t-test compare. Which test did you use for the categorical data such as sex and blood type?

**VR. We thank the reviewer for this helpful suggestion. The distribution of continuous variables (age, weight, and body temperature) was assessed using graphical diagnostics (Q–Q plots and histograms). Because of the relatively small sample size and potential unequal variances between groups, Welch’s t-test was used for group comparisons. Following the reviewer’s recommendation, median and interquartile range (IQR) values have now been added to Supplementary Table S2 to better illustrate data distribution. For categorical variables (sex and blood group), Fisher’s exact test was applied due to the small sample size. The statistical tests used are now indicated in Supplementary Table S2 and clarified in the table legend.**

- Supplementary figures 2, 3, 4, and 5: “value” is not an acceptable Y-axis label, you have the proper labels in the figure legends, please add them on each figure plot. Please add the statistical test on each plot if the p-value is significant. As of now, we don’t know how to interpret these results.

**VR. We thank the reviewer for this helpful comment. The generic Y-axis label “Value” has now been replaced with the appropriate measurement labels in all revised supplementary figures. Because each panel represents a different parameter measured in different units, the corresponding units are now indicated in the figure legends. Following the reviewer’s suggestion, statistical significance annotations (asterisks) have also been added directly to the relevant panels for parameters with P < 0.05, while the exact P-values remain reported in the corresponding tables. These revisions improve the clarity and interpretation of the supplementary figures.**

- For the supplemental figures 3 and 5, please indicate if the data are coming from the plasma or from the supernatant of the in vitro stimulation assay.

**VR. We thank the reviewer for this helpful suggestion. The legends of the supplementary figures have been revised to explicitly indicate the biological source of the cytokine and chemokine measurements. Specifically, we now state that cytokine and chemokine levels shown in these figures were quantified from circulating plasma samples using a Luminex multiplex assay. These clarifications have been added to the figure legends to avoid any ambiguity regarding the origin of the measured immune parameters.**

- Supplemental figure legend 1 and 3: What do you mean by “values are normalized residuals; negatives indicate below-average levels”? (line 47-48), please indicate any transformation of the data in the methods and/or figure legends.

**VR. We thank the reviewer for pointing out this lack of clarity. The wording “normalized residuals” was inaccurate and has been corrected. The values shown in these figures correspond to z-score standardized variables, obtained by centering each variable (subtracting the mean) and scaling by its standard deviation to allow comparison of parameters measured on different scales. The legends of Supplementary Figures 1 and 3 have been revised accordingly, and the transformation is now explicitly described in the Methods section.**

- Supplemental figure legend 2 and 4: Please differentiate data coming from the plasma and the ones coming from the supernatant of the in vitro stimulation. I do not understand “values are normalized residuals; negatives indicate below-average levels” in this section. Luminex kit outcomes from cytokines/chemokines kits are quantitative based on the standards provided by the kit. Here you mention MFI instead of the analyzed data from the standard curve. Did you have issues with your standards? Even using the MFI data, what do you mean by “values are normalized residuals; negatives indicate below-average levels”? What did you do to the data to transform them? It is highly unclear.

**VR. We thank the reviewer for this important comment and agree that the previous wording was unclear. The legends of Supplementary Figures 2 and 4 have been revised to clarify that the cytokine and chemokine measurements shown correspond to circulating plasma samples, and not to supernatants from PBMC stimulation assays. Cytokine levels were quantified using a Luminex multiplex assay. Because several analytes were close to the lower detection range, analyses were performed using background-adjusted median fluorescence intensity (MFI) values rather than concentrations derived from the standard curves. No issues were observed with the assay standards. In addition, the phrase “normalized residuals” was incorrect and has been replaced. The plotted values correspond to z-score standardized measurements (mean-centered and scaled by the standard deviation) to allow comparison across variables measured on different scales. The Methods section and figure legends have been revised accordingly.**

**Reviewer #2:**

1. The results section should begin with a more clear description of the sampled animals akin to a cross-sectional study of humans that includes not just age range and sex, but also living conditions and prevalence of malaria in surrounding areas.

**VR. We thank the reviewer for this helpful suggestion. In the revised manuscript, we have added a short paragraph at the beginning of the Results section describing the study population. This paragraph now summarizes the number of chimpanzees sampled, their sex composition, age range, and shared environmental context. We also clarify that all individuals originated from the same geographical region in Gabon and were sampled under comparable conditions, and that malaria parasites circulate endemically in wild African ape populations in this region. This information provides the ecological and epidemiological background for interpreting the cross-sectional analysis.**

2. The breakdown of infections in each site is important for Figure 1

**VR. We thank the reviewer for this helpful suggestion. Figure 1A has been revised to explicitly show the distribution of infection categories across the two sampling sites. The updated bar plot now displays the prevalence of each infection category in the full cohort as well as the corresponding site-specific prevalence for the two locations (CIRMF and La Lékédi Park). Distinct bar patterns are used to distinguish the two sites, allowing readers to directly visualize how infection categories are distributed across sampling locations.**

3. Line 168: When comparing 2/3 groups, and 2/2 groups with more than one data point, it doesn’t make sense to say the mixed infections are different than the one monoinfection.

**VR. We thank the reviewer for highlighting this point. The *P. ovale-like* infection was detected in only a single individual (n = 1), and therefore this case was not included in statistical comparisons. In the revised manuscript we clarified this explicitly in the Results section, indicating that the PO case is presented only for descriptive purposes ‘lines 252-255). All statistical analyses comparing infection groups were conducted only between groups with sufficient sample size (PG/PR, PG/PR/PV, and non-infected individuals).**

4. Line 189 phrase “emphasized some less clear species-specific patterns.” Doesn’t make sense

**VR. We thank the reviewer for pointing out the lack of clarity in this sentence. The wording has been revised to better reflect the PCA results. The sentence now reads: *“Stratification by infection group (PG/PR, PG/PR/PV, PO) revealed only weak species-specific patterns.”*  (lines 252-255). We then clarify that PCA based on blood parameters did not show clear clustering between groups, whereas cytokine/chemokine PCA showed partial separation of PG/PR-infected animals from non-infected individuals along PC2 (Figure 2D).**

5. Please add stats in Figure 3

**VR. We thank the reviewer for this suggestion. Statistical significance annotations have now been added directly to the relevant panels in Figure 3 to facilitate interpretation of the results. Asterisks indicate statistically significant differences between groups (*P < 0.05; **P < 0.01). The exact P-values and corresponding statistical tests remain reported in Tables 1 and 2. The figure legend has been updated accordingly.**
