# Supplementary material for: Hidden hematological, biochemical and immune costs of asymptomatic malaria infections in semi-wild chimpanzees
Source: PLoS Pathog. 2026 Jun 23;22(6):e1014287. doi: 10.1371/journal.ppat.1014287 (PMC13289926; doi:10.1371/journal.ppat.1014287)
Supplement: Attachment — Submitted filename: Answers_Editor_Reviewers_PlosPat_17May2026.docx [file ppat.1014287.s008.docx]

**REHABS International Research Laboratory**

**Nelson Mandela University**

**George Campus, Madiba Drive,**

**Response to reviewers – 17^th^ May 2026**

We sincerely thank the editor and the two reviewers for the final careful evaluation of our manuscript and for the constructive suggestions provided. We are pleased that the manuscript is considered close to acceptance. Following the reviewer’s final recommendation, we have revised the manuscript to moderate and clarify the interpretation regarding age-related patterns of parasitemia and infection.

**Reviewer #2 comment.** “The statements about higher parasitemia in younger animals should likely be removed or significantly restated given the lack of statistics in Fig. 1B and lack of correlation in Fig. 1D. As it stands, the data could be summarized as ‘we did not observe a significant correlation between age and parasitemia. However, this should be interpreted with caution as only two animals were <7 years old, only one of which was infected.’ If discussed, it should be placed within context of humans where the primary burden of acute and chronic infection is in school-aged children and infants.”

**VR.** We thank the reviewer for this clarification and agree that the interpretation of age-related parasitemia patterns required a more cautious interpretation. In the revised manuscript, we moderated the wording throughout the Results, Discussion, Abstract, and Author Summary to avoid overinterpreting the relationship between age and parasitemia. Specifically, we removed statements suggesting a significant association between younger age and higher parasitemia. The Results section now clearly states that no significant correlation was detected between age and parasitemia and emphasizes that interpretation of this relationship is limited by the age structure of the cohort, which included only two individuals younger than seven years old, only one of whom was infected. In addition, the Discussion now places these observations within the broader context of human malaria epidemiology, where the highest burden of infection and disease is typically observed in infants and school-aged children prior to the acquisition of partial immunity through repeated exposure. We now explicitly state that whether similar age-dependent infection dynamics occur in African great apes remains unresolved and will require larger longitudinal studies including a better representation of juvenile individuals. The corresponding revisions were made in the Abstract, Author Summary, Results section (“Age impact on *Plasmodium* prevalence and multi-species infections consequences on parasitemia in chimpanzees”), and Discussion, and all modifications have been highlighted in yellow in the revised manuscript.
