# Supplementary material for: Hidden hematological, biochemical and immune costs of asymptomatic malaria infections in semi-wild chimpanzees
Source: PLoS Pathog. 2026 Jun 23;22(6):e1014287. doi: 10.1371/journal.ppat.1014287 (PMC13289926; doi:10.1371/journal.ppat.1014287)
Supplement: Peer review history

# Peer review: Decision Letter 0

**DOI:** 10.1371/journal.ppat.1014287.r001

**Authors:** Margaret Phillips, James Beeson

23 Dec 2025

-->PPATHOGENS-D-25-02784

Hidden hematological, biochemical and immune costs of asymptomatic malaria infections in semi-wild chimpanzees

PLOS Pathogens

Dear Dr. Rougeron,

Thank you for submitting your manuscript to PLOS Pathogens. After careful consideration, we feel that it has merit but does not fully meet PLOS Pathogens's publication criteria as it currently stands. Therefore, we invite you to submit a revised version of the manuscript that addresses the points raised during the review process.

Please submit your revised manuscript by Feb 21 2026 11:59PM. If you will need more time than this to complete your revisions, please reply to this message or contact the journal office at plospathogens@plos.org. When you're ready to submit your revision, log on to https://www.editorialmanager.com/ppathogens/ and select the 'Submissions Needing Revision' folder to locate your manuscript file.

Please include the following items when submitting your revised manuscript:

* A letter that responds to each point raised by the editor and reviewer(s). You should upload this letter as a separate file labeled 'Response to Reviewers'. This file does not need to include responses to any formatting updates and technical items listed in the 'Journal Requirements' section below.

* A marked-up copy of your manuscript that highlights changes made to the original version. You should upload this as a separate file labeled 'Revised Manuscript with Track Changes'.

* An unmarked version of your revised paper without tracked changes. You should upload this as a separate file labeled 'Manuscript'.

If you would like to make changes to your financial disclosure, competing interests statement, or data availability statement, please make these updates within the submission form at the time of resubmission. Guidelines for resubmitting your figure files are available below the reviewer comments at the end of this letter.

We look forward to receiving your revised manuscript.

Kind regards,

James G. Beeson, MBBS, PhD

Academic Editor

PLOS Pathogens

Margaret Phillips

Section Editor

PLOS Pathogens

Sumita Bhaduri-McIntosh

Editor-in-Chief

PLOS Pathogens

orcid.org/0000-0003-2946-9497

Michael Malim

-->Editor-in-Chief

PLOS Pathogens

orcid.org/0000-0002-7699-2064

**Additional Editor Comments:**

The two reviewers have provided many helpful and thoughtful comments and suggestions for improvement of your manuscript. Please address all the reviewers comments as best you can, and you can contact me if you have any concerns or queries.

**Journal Requirements:**

If the reviewer comments include a recommendation to cite specific previously published works, please review and evaluate these publications to determine whether they are relevant and should be cited. There is no requirement to cite these works unless the editor has indicated otherwise.

1) We ask that a manuscript source file is provided at Revision. Please upload your manuscript file as a .doc, .docx, .rtf or .tex. If you are providing a .tex file, please upload it under the item type u2018LaTeX Source Fileu2019 and leave your .pdf version as the item type u2018Manuscriptu2019.

2) Please provide an Author Summary. This should appear in your manuscript between the Abstract (if applicable) and the Introduction, and should be 150-200 words long. The aim should be to make your findings accessible to a wide audience that includes both scientists and non-scientists. Sample summaries can be found on our website under Submission Guidelines:

https://journals.plos.org/plospathogens/s/submission-guidelines#loc-parts-of-a-submission

3) We noticed that you used the phrase 'unpublished data' in the manuscript. We do not allow these references, as the PLOS data access policy requires that all data be either published with the manuscript or made available in a publicly accessible database. Please amend the supplementary material to include the referenced data or remove the references.

4) We do not publish any copyright or trademark symbols that usually accompany proprietary names, eg ©,  ®, or TM  (e.g. next to drug or reagent names). Therefore please remove all instances of trademark/copyright symbols throughout the text, including:

- © on page: 17

- ® on pages: 14, and 16.

5) Please upload all main figures as separate Figure files in .tif or .eps format. For more information about how to convert and format your figure files please see our guidelines:

https://journals.plos.org/plospathogens/s/figures

6) We have noticed that you have uploaded Supporting Information files, but you have not included a list of legends. Please add a full list of legends for your Supporting Information files after the references list.

7) Some material included in your submission may be copyrighted. According to PLOSu2019s copyright policy, authors who use figures or other material (e.g., graphics, clipart, maps) from another author or copyright holder must demonstrate or obtain permission to publish this material under the Creative Commons Attribution 4.0 International (CC BY 4.0) License used by PLOS journals. Please closely review the details of PLOSu2019s copyright requirements here: PLOS Licenses and Copyright. If you need to request permissions from a copyright holder, you may use PLOS's Copyright Content Permission form.

Please respond directly to this email and provide any known details concerning your material's license terms and permissions required for reuse, even if you have not yet obtained copyright permissions or are unsure of your material's copyright compatibility. Once you have responded and addressed all other outstanding technical requirements, you may resubmit your manuscript within Editorial Manager.

Potential Copyright Issues:

i) Figure 5. Please confirm whether you drew the images / clip-art within the figure panels by hand. If you did not draw the images, please provide (a) a link to the source of the images or icons and their license / terms of use; or (b) written permission from the copyright holder to publish the images or icons under our CC BY 4.0 license. Alternatively, you may replace the images with open source alternatives. See these open source resources you may use to replace images / clip-art:

- https://commons.wikimedia.org

- https://openclipart.org/.

ii) Figure 1A. Please (a) provide a direct link to the base layer of the map (i.e., the country or region border shape) and ensure this is also included in the figure legend; and (b) provide a link to the terms of use / license information for the base layer image or shapefile. We cannot publish proprietary or copyrighted maps (e.g. Google Maps, Mapquest) and the terms of use for your map base layer must be compatible with our CC BY 4.0 license.

Note: if you created the map in a software program like R or ArcGIS, please locate and indicate the source of the basemap shapefile onto which data has been plotted.

If your map was obtained from a copyrighted source please amend the figure so that the base map used is from an openly available source. Alternatively, please provide explicit written permission from the copyright holder granting you the right to publish the material under our CC BY 4.0 license.

If you are unsure whether you can use a map or not, please do reach out and we will be able to help you. The following websites are good examples of where you can source open access or public domain maps:

* U.S. Geological Survey (USGS) - All maps are in the public domain. (http://www.usgs.gov)

* PlaniGlobe - All maps are published under a Creative Commons license so please cite u201cPlaniGlobe, http://www.planiglobe.com, CC BY 2.0u201d in the image credit after the caption. (http://www.planiglobe.com/?lang=enl)

* Natural Earth - All maps are public domain. (http://www.naturalearthdata.com/about/terms-of-use/).

8) In the online submission form, you indicated that  The raw data supporting the conclusions of this article is available upon request.. All PLOS journals now require all data underlying the findings described in their manuscript to be freely available to other researchers, either

1. In a public repository

2. Within the manuscript itself

3. Uploaded as supplementary information.

This policy applies to all data except where public deposition would breach compliance with the protocol approved by your research ethics board. If your data cannot be made publicly available for ethical or legal reasons (e.g., public availability would compromise patient privacy), please explain your reasons by return email and your exemption request will be escalated to the editor for approval. Your exemption request will be handled independently and will not hold up the peer review process, but will need to be resolved should your manuscript be accepted for publication. One of the Editorial team will then be in touch if there are any issues.

9) Kindly revise your competing statement in the online submission form to align with the journal's style guidelines: 'The authors declare that there are no competing interests.'

**Reviewers' Comments:**

Reviewer's Responses to Questions

**Part I - Summary**

Please use this section to discuss strengths/weaknesses of study, novelty/significance, general execution and scholarship.

Reviewer #1: The study by Nowakowski highlights very important and interesting points, as great apes can be infected by malaria parasites, including some species similar to those that infect humans, if I understood correctly. A better understanding of malaria infection in great apes, as demonstrated in the work presented here, is essential to achieving the ultimate goal of eradicating malaria. However, the presentation of the study requires greater rigor, specifically regarding the Luminex data and the statistical tests used. Currently, the methods section lacks sufficient information to allow for reproducibility of the study. I advise the authors to assess the distribution of their data to determine whether the use of parametric tests is appropriate. If this analysis has already been performed, the information should be included in the methods. Regarding the Luminex data, it is unclear which samples were used in each of the two different Luminex kits, and which reported data are from plasma samples versus PBMC-stimulated supernatants. Clarifications on these points will greatly strengthen the manuscript.

Reviewer #2: The authors present an excellent piece of work representing a unique opportunity to rigorously assess Plasmodium infection in chimpanzees. The conclusions are generally well aligned with the data, and the work is very nearly complete. The authors should be commended on an ambitious and fascinating project. There are a couple deficits which should be corrected to enable full realization of the work including: 1) clarifying true negativity in the uninfected group and; 2) drawing clearer comparisons to human infections in terms of age distribution and acquisition of asymptomatic infection.

**********

**Part II – Major Issues: Key Experiments Required for Acceptance**

Please use this section to detail the key new experiments or modifications of existing experiments that should be absolutely required to validate study conclusions.

Generally, there should be no more than 3 such required experiments or major modifications for a "Major Revision" recommendation. If more than 3 experiments are necessary to validate the study conclusions, then you are encouraged to recommend "Reject".

Reviewer #1: Major issues:

- The Luminex assays are not clear. You mention 2 kits (one from Invitrogen and one from BioTechne), are they different? If yes, do you mean that you use one kit for the plasma sample and the other one for the supernatant of the in vitro stimulation assay? Can you please give the proper reference to these kits? As of now, using only the name you cited is not enough to find a kit. Did you compare data generated from these 2 kits? How did you correct for possible batch effect? In the manuscript, sometimes you mentioned MFI and sometimes quantitative data. Can you please expand on how the data from these 2 kits were analyze and if any quantitative analysis were performed, how you did. Also, how much volume did you use? at which dilutions? Singlet or duplicate? Luminex kits usually use different dilution of the plasma versus the supernatants. As of now, we do not know where the data shown in the figures are coming from (plasma vs supernatant). It is crucial to better indicate which analysis you did using the plasma data versus the supernatants from the PMA/Iono stimulations of the PBMCs as is can mislead the interpretation.

- Welch’s test is a parametric test. Did you assess the distribution of the data prior selecting parametric test? If not, you should assess the distribution of your data prior selecting a test. Data that are not following a normal distribution should use a non-parametric test. I will suggest to the authors to review all their data distribution and tests. Line 517-519: What do you mean by “standardized using z-scores”? what did you standardized exactly? Please add details.

Reviewer #2: 1. Can the authors include qPCR data for the nanopore-negative samples as well? This will not only help ensure accuracy of the nanopore, but strengthen arguments about asymptomatic infection.

2. For the blood parameters in Tables 1 and 2 as well as Figure 3, could the authors provide some context of normal ranges. I understand these may be speculative for chimpanzees, but rough reference values should be available. This will help interpret what, if any, of these deviations are clinically significant.

3. The effect of age is a very interesting and important finding, yet needs to be more concrete. Since there is only a single animal under ~7 years of age, this should be a major caveat discussed considering that the heaviest burden of infection and disease in humans occurs in the first few years of life. Secondly, to claim that the older animals are uninfected, qPCR would be needed.

4. The unpublished longitudinal data mentioned in the Discussion should either be presented to add context or removed.

5. Non-human primates, at least those in captive colonies, mask symptoms and pain extremely well especially in the presence of humans. Considering this and that animals were sedated during collection, discussion of “asymptomatic” infection needs to be adjusted and supported by more data. In humans, symptomatic malaria includes positivity (often by smear) and fever, chills, etc. Obviously, symptoms outside of fever are impossible to assess. Therefore, it would be more useful if temperature is included (if measured) and the discussion centered around comparisons of objective values obtained from blood paramaters to not just normal ranges but also those seen for malaria infections in human across the ages (from symptomatic children to asymptomatic adults). This is done to some extent for the inflammatory cytokines, but more context via comparisons to healthy and unhealthy values would greatly improve interpretability.

**********

**Part III – Minor Issues: Editorial and Data Presentation Modifications**

Please use this section for editorial suggestions as well as relatively minor modifications of existing data that would enhance clarity.

Reviewer #1: Introduction:

- Are these plasmodium species carried by the same vector than the one infecting human?

- Do you consider the great apes as potential reservoir for malaria?

- Did you find similar species in the Plasmodium infecting Great Apes and Human in Gabon?

Methods:

- For how many individuals the CBC was unavailable?

- PBMCs stimulation section: how many cells did you stimulate per individuals? And in which final volume of culture media? This information matters as you are using the supernatant to assess the cytokines/chemokines. Adding these details will allow the reproducibility of your data.

- Line 501-502: cytokines/chemokines were assessed from the plasma sample and the supernatants of the stimulated PBMCs. Please correct as of now it sounds that you tested plasma from stimulated PBMCs which is not what it is.

- Data availability: you should consider sharing your Luminex and other data through the ImmPort portal.

Results:

- It is not clear how many chimpanzees were infected versus non-infected and how this determination was made: qPCR results only? Or did you include other parameters such as anemia, fever?

- The tables will benefit to have the age (median/IQR) for each compared set of groups as it seems from the figure 1B that most of the non-infected are older than the infected ones.

- Did you correct by age for the analysis as it seems to be a cofounding factor?

- It will help to know the number of individuals for each group (PG/PR, PO, …), the only information is 1 individual with Po.

- Figure 3, it is not clear if the cytokines/chemokines data are coming from the plasma or from the supernatants of the PBMCs in vitro stimulation assay. Did the authors compare the levels of cytokines/chemokines between the plasma of infected and non-infected individuals, but also between the plasma and supernatants for each group? Because PMA/Ionomycin is a “hammer: triggering high levels of cytokines responses it is not malaria specific. However, comparing the data from the plasma versus the in vitro assay will allow to better be found correlation between the cytokines that are specifically induced during malaria infections.

- Missing Y-axis on all the figure 3

- Figure 4C, what means “value” in the Y-axis?

- The figures will benefit to have the statistical test outcomes indicated.

Discussion:

- Line 246: You mentioned that having more malaria infection in youngling vs adults in chimpanzees’ contrast with human malaria. However, in human, malaria (symptomatic and asymptomatic) is found more frequently in children living in high malaria transmission area compared to the adults. I am not sure I am getting your point. Can you develop your thoughts?

- Line 289: It is complicated to make a point with “unpublished data” or “data not shown”. Is it possible to add a figure with this data?

- In your interpretation of the cytokines/chemokines, please indicate where these data come from, the plasma or the stimulated PBMCs, as they are not bringing the same type of information which can mislead the interpretation.

Supplementary:

- Supplementary table 1: why there are only the results for 13 individuals? Were they 13 individuals infected with malaria and 14 individuals non-infected (total of 27 individuals)? They are no individuals’ names followed by an asterisk; does it mean that none of them were infected with P. vivax-like? Is it based on this assay that you categorized your “infected” vs “non-infected group”? If so, please add a section about it in the methods.

- Supplementary table 2: Please indicate if you assessed the distribution of your data to justify the use of the t-test (which one did you used?). The table will benefit of having the median (IQR) values to show the distribution of the data that the t-test compare. Which test did you use for the categorical data such as sex and blood type?

- Supplementary figures 2, 3, 4, and 5: “value” is not an acceptable Y-axis label, you have the proper labels in the figure legends, please add them on each figure plot. Please add the statistical test on each plot if the p-value is significant. As of now, we don’t know how to interpret these results.

- For the supplemental figures 3 and 5, please indicate if the data are coming from the plasma or from the supernatant of the in vitro stimulation assay.

- Supplemental figure legend 1 and 3: What do you mean by “values are normalized residuals; negatives indicate below-average levels”? (line 47-48), please indicate any transformation of the data in the methods and/or figure legends.

- Supplemental figure legend 2 and 4: Please differentiate data coming from the plasma and the ones coming from the supernatant of the in vitro stimulation. I do not understand “values are normalized residuals; negatives indicate below-average levels” in this section. Luminex kit outcomes from cytokines/chemokines kits are quantitative based on the standards provided by the kit. Here you mention MFI instead of the analyzed data from the standard curve. Did you have issues with your standards? Even using the MFI data, what do you mean by “values are normalized residuals; negatives indicate below-average levels”? What did you do to the data to transform them? It is highly unclear.

Reviewer #2: 1. The results section should begin with a more clear description of the sampled animals akin to a cross-sectional study of humans that includes not just age range and sex, but also living conditions and prevalence of malaria in surrounding areas.

2. The breakdown of infections in each site is important for Figure 1

3. Line 168: When comparing 2/3 groups, and 2/2 groups with more than one data point, it doesn’t make sense to say the mixed infections are different than the one monoinfection.

4. Line 189 phrase “emphasized some less clear species-specific patterns.” Doesn’t make sense

5. Please add stats in Figure 3

**********

PLOS authors have the option to publish the peer review history of their article (what does this mean?). If published, this will include your full peer review and any attached files.

If you choose “no”, your identity will remain anonymous but your review may still be made public.

**Do you want your identity to be public for this peer review?**  For information about this choice, including consent withdrawal, please see our Privacy Policy.

Reviewer #1: **Yes:**  Catherine S. Forconi

Reviewer #2: **Yes:**  Brandon K. Wilder

**Figure resubmission:**

-->While revising your submission, we strongly recommend that you use PLOS’s NAAS tool (https://ngplosjournals.pagemajik.ai/artanalysis) to test your figure files. NAAS can convert your figure files to the TIFF file type and meet basic requirements (such as print size, resolution), or provide you with a report on issues that do not meet our requirements and that NAAS cannot fix.

After uploading your figures to PLOS’s NAAS tool - https://ngplosjournals.pagemajik.ai/artanalysis, NAAS will process the files provided and display the results in the "Uploaded Files" section of the page as the processing is complete. If the uploaded figures meet our requirements (or NAAS is able to fix the files to meet our requirements), the figure will be marked as "fixed" above. If NAAS is unable to fix the files, a red "failed" label will appear above. When NAAS has confirmed that the figure files meet our requirements, please download the file via the download option, and include these NAAS processed figure files when submitting your revised manuscript.

**Reproducibility:**

To enhance the reproducibility of your results, we recommend that authors of applicable studies deposit laboratory protocols in protocols.io, where a protocol can be assigned its own identifier (DOI) such that it can be cited independently in the future. Additionally, PLOS ONE offers an option to publish peer-reviewed clinical study protocols. Read more information on sharing protocols at https://plos.org/protocols?utm_medium=editorial-email&utm_source=authorletters&utm_campaign=protocols

# Author comment: Author response to Decision Letter 1

**DOI:** 10.1371/journal.ppat.1014287.r002

18 Mar 2026

Attachment Submitted filename: Answers_Editor_Reviewers_PlosPat_2026.docx

# Peer review: Decision Letter 1

**DOI:** 10.1371/journal.ppat.1014287.r003

**Authors:** Margaret Phillips, James Beeson, Margaret Phillips, James Beeson

15 May 2026

PPATHOGENS-D-25-02784R1

Hidden hematological, biochemical and immune costs of asymptomatic malaria infections in semi-wild chimpanzees

PLOS Pathogens

Dear Dr. Rougeron,

Thank you for submitting your manuscript to PLOS Pathogens. After careful consideration, we believe your paper is now very close to being accepted, pending some final minor revisions. Therefore, we invite you to submit a revised version of the manuscript that addresses the final minor points raised during the review process.

Please submit your revised manuscript by Jul 14 2026 11:59PM. If you will need more time than this to complete your revisions, please reply to this message or contact the journal office at plospathogens@plos.org. When you're ready to submit your revision, log on to https://www.editorialmanager.com/ppathogens/ and select the 'Submissions Needing Revision' folder to locate your manuscript file.

Please include the following items when submitting your revised manuscript:

* A letter that responds to each point raised by the editor and reviewer(s). You should upload this letter as a separate file labeled 'Response to Reviewers'. This file does not need to include responses to any formatting updates and technical items listed in the 'Journal Requirements' section below.

* A marked-up copy of your manuscript that highlights changes made to the original version. You should upload this as a separate file labeled 'Revised Manuscript with Track Changes'.

* An unmarked version of your revised paper without tracked changes. You should upload this as a separate file labeled 'Manuscript'.

If you would like to make changes to your financial disclosure, competing interests statement, or data availability statement, please make these updates within the submission form at the time of resubmission. Guidelines for resubmitting your figure files are available below the reviewer comments at the end of this letter.

As the corresponding author, your ORCID iD is verified in the submission system and will appear in the published article. PLOS supports the use of ORCID, and we encourage all coauthors to register for an ORCID iD and use it as well. Please encourage your coauthors to verify their ORCID iD within the submission system before final acceptance, as unverified ORCID iDs will not appear in the published article. Only the individual author can complete the verification step; PLOS staff cannot verify ORCID iDs on behalf of authors.

We look forward to receiving your revised manuscript.

Kind regards,

James G. Beeson, MBBS, PhD

Academic Editor

PLOS Pathogens

Margaret Phillips

Section Editor

PLOS Pathogens

Sumita Bhaduri-McIntosh

Editor-in-Chief

PLOS Pathogens

orcid.org/0000-0003-2946-9497

Michael Malim

Editor-in-Chief

PLOS Pathogens

orcid.org/0000-0002-7699-2064

**Additional Editor Comments:**

Thank you for addressing the reviewers comments. Please address the final minor revisions requested from reviewer 2.

After you have completed this, I believe your paper will be ready to progress to formal acceptance.

**Reviewers' Comments:**

Reviewer's Responses to Questions

**Part I - Summary**

Please use this section to discuss strengths/weaknesses of study, novelty/significance, general execution and scholarship.

Reviewer #1: All comments have been addressed. The conclusions are well supported by the results of this study. The methods are now clear and reproducible.

Reviewer #2: Thank you to the reviewers for taking the time to significantly edit their manuscript. The work remains as important and is now presented more clearly.

**********

**Part II – Major Issues: Key Experiments Required for Acceptance**

Please use this section to detail the key new experiments or modifications of existing experiments that should be absolutely required to validate study conclusions.

Generally, there should be no more than 3 such required experiments or major modifications for a "Major Revision" recommendation. If more than 3 experiments are necessary to validate the study conclusions, then you are encouraged to recommend "Reject".

Reviewer #1: (No Response)

Reviewer #2: None.

**********

**Part III – Minor Issues: Editorial and Data Presentation Modifications**

Please use this section for editorial suggestions as well as relatively minor modifications of existing data that would enhance clarity.

Reviewer #1: (No Response)

Reviewer #2: The statements about higher parasitemia in younger animals should likely be removed or significantly restated given the lack of statstics in Fig. 1B and lack of correlation in Fig. 1D. As it stands, the data could be summarized as “we did not not observe a significant correlation between age and parasitemia. However, this should be interpreted with caution only two animals were <7 years old, only one of which was infected.” If discussed, it should be placed within context of human where the primary burden of acute and chronic infection is in school-aged children and infants.

**********

PLOS authors have the option to publish the peer review history of their article (what does this mean?). If published, this will include your full peer review and any attached files.

If you choose “no”, your identity will remain anonymous but your review may still be made public.

**Do you want your identity to be public for this peer review?**  For information about this choice, including consent withdrawal, please see our Privacy Policy.

Reviewer #1: **Yes:**  Catherine S. Forconi

Reviewer #2: **Yes:**  Brandon Wilder

[NOTE: If reviewer comments were submitted as an attachment file, they will be attached to this email and accessible via the submission site. Please log into your account, locate the manuscript record, and check for the action link "View Attachments". If this link does not appear, there are no attachment files.]

**Figure resubmission:**

-->While revising your submission, we strongly recommend that you use PLOS’s NAAS tool (https://ngplosjournals.pagemajik.ai/artanalysis) to test your figure files. NAAS can convert your figure files to the TIFF file type and meet basic requirements (such as print size, resolution), or provide you with a report on issues that do not meet our requirements and that NAAS cannot fix.

After uploading your figures to PLOS’s NAAS tool - https://ngplosjournals.pagemajik.ai/artanalysis, NAAS will process the files provided and display the results in the "Uploaded Files" section of the page as the processing is complete. If the uploaded figures meet our requirements (or NAAS is able to fix the files to meet our requirements), the figure will be marked as "fixed" above. If NAAS is unable to fix the files, a red "failed" label will appear above. When NAAS has confirmed that the figure files meet our requirements, please download the file via the download option, and include these NAAS processed figure files when submitting your revised manuscript.

**Reproducibility:**

To enhance the reproducibility of your results, we recommend that authors of applicable studies deposit laboratory protocols in protocols.io, where a protocol can be assigned its own identifier (DOI) such that it can be cited independently in the future. Additionally, PLOS ONE offers an option to publish peer-reviewed clinical study protocols. Read more information on sharing protocols at https://plos.org/protocols?utm_medium=editorial-email&utm_source=authorletters&utm_campaign=protocols

# Author comment: Author response to Decision Letter 2

**DOI:** 10.1371/journal.ppat.1014287.r004

19 May 2026

Attachment Submitted filename: Answers_Editor_Reviewers_PlosPat_17May2026.docx

# Editor report: Decision Letter 2

**DOI:** 10.1371/journal.ppat.1014287.r005

**Authors:** Margaret Phillips, James Beeson, Margaret Phillips, James Beeson, Margaret Phillips, James Beeson

21 May 2026

Dear Dr. Rougeron,

We are pleased to inform you that your manuscript 'Hidden hematological, biochemical and immune costs of asymptomatic malaria infections in semi-wild chimpanzees' has been provisionally accepted for publication in PLOS Pathogens.

Before your manuscript can be formally accepted you will need to complete some formatting changes, which you will receive in a follow up email. A member of our team will be in touch with a set of requests.

Please note that your manuscript will not be scheduled for publication until you have made the required changes, so a swift response is appreciated.

IMPORTANT: The editorial review process is now complete. PLOS will only permit corrections to spelling, formatting or significant scientific errors from this point onwards. Requests for major changes, or any which affect the scientific understanding of your work, will cause delays to the publication date of your manuscript.

Should you, your institution's press office or the journal office choose to press release your paper, you will automatically be opted out of early publication. We ask that you notify us now if you or your institution is planning to press release the article. All press must be co-ordinated with PLOS.

Thank you again for supporting Open Access publishing; we are looking forward to publishing your work in PLOS Pathogens.

Best regards,

James G. Beeson, MBBS, PhD

Academic Editor

PLOS Pathogens

Margaret Phillips

Section Editor

PLOS Pathogens

Sumita Bhaduri-McIntosh

Editor-in-Chief

PLOS Pathogens

orcid.org/0000-0003-2946-9497

Michael Malim

Editor-in-Chief

PLOS Pathogens

orcid.org/0000-0002-7699-2064

***********************************************************

Reviewer Comments (if any, and for reference):

# Editor report: Acceptance letter

**DOI:** 10.1371/journal.ppat.1014287.r006

**Authors:** Margaret Phillips, James Beeson, Margaret Phillips, James Beeson, Margaret Phillips, James Beeson

Dear Dr. Rougeron,

We are delighted to inform you that your manuscript, "Hidden hematological, biochemical and immune costs of asymptomatic malaria infections in semi-wild chimpanzees," has been formally accepted for publication in PLOS Pathogens.

We have now passed your article onto the PLOS Production Department who will complete the rest of the pre-publication process. All authors will receive a confirmation email upon publication.

The corresponding author will soon be receiving a typeset proof for review, to ensure errors have not been introduced during production. Please review the PDF proof of your manuscript carefully, as this is the last chance to correct any scientific or type-setting errors. Please note that major changes, or those which affect the scientific understanding of the work, will likely cause delays to the publication date of your manuscript. Note: Proofs for Front Matter articles (Pearls, Reviews, Opinions, etc...) are generated on a different schedule and may not be made available as quickly.

Soon after your final files are uploaded, the early version of your manuscript, if you opted to have an early version of your article, will be published online. The date of the early version will be your article's publication date. The final article will be published to the same URL, and all versions of the paper will be accessible to readers.

For Research Articles, you will receive an invoice from PLOS for your publication fee after your manuscript has reached the completed accept phase. If you receive an email requesting payment before acceptance or for any other service, this may be a phishing scheme. Learn how to identify phishing emails and protect your accounts at https://explore.plos.org/phishing.

Thank you again for supporting open-access publishing; we are looking forward to publishing your work in PLOS Pathogens.

Best regards,

Sumita Bhaduri-McIntosh

Editor-in-Chief

PLOS Pathogens

orcid.org/0000-0003-2946-9497

Michael Malim

Editor-in-Chief

PLOS Pathogens

orcid.org/0000-0002-7699-2064
